# Time-Aware Multi-Agent Symbiosis

**DOI:** 10.3389/frobt.2020.503452

**Published:** 2020-11-12

**Authors:** Michail Maniadakis, Emmanouil Hourdakis, Markos Sigalas, Stylianos Piperakis, Maria Koskinopoulou, Panos Trahanias

**Affiliations:** Institute of Computer Science, Foundation for Research and Technology – Hellas (FORTH), Heraklion, Greece

**Keywords:** human robot interaction (HRI), artificial time perception, eterogeneous multi-agent planning, autonomous systems, collaborative task execution

## Abstract

Contemporary research in human-machine symbiosis has mainly concentrated on enhancing relevant sensory, perceptual, and motor capacities, assuming short-term and nearly momentary interaction sessions. Still, human-machine confluence encompasses an inherent temporal dimension that is typically overlooked. The present work shifts the focus on the temporal and long-lasting aspects of symbiotic human-robot interaction (sHRI). We explore the integration of three time-aware modules, each one focusing on a diverse part of the sHRI timeline. Specifically, the Episodic Memory considers past experiences, the Generative Time Models estimate the progress of ongoing activities, and the Daisy Planner devices plans for the timely accomplishment of goals. The integrated system is employed to coordinate the activities of a multi-agent team. Accordingly, the proposed system (i) predicts human preferences based on past experience, (ii) estimates performance profile and task completion time, by monitoring human activity, and (iii) dynamically adapts multi-agent activity plans to changes in expectation and Human-Robot Interaction (HRI) performance. The system is deployed and extensively assessed in real-world and simulated environments. The obtained results suggest that building upon the unfolding and the temporal properties of team tasks can significantly enhance the fluency of sHRI.

## 1. Introduction

Fluent, symbiotic Human-Robot Interaction (sHRI) is an important, yet challenging problem in robotics research as evidenced by the increasing number of published works (Rosenthal et al., 2010; Fernando et al., 2014; Liu et al., 2016; Riccio et al., 2016) and review papers (Coradeschi and Loutfi, 2008; Green et al., 2008; Carrillo and Topp, 2016; Tsarouchi et al., 2016). Despite the significant resources devoted in sHRI, the majority of existing systems consider mainly the spatial aspects of the world without encapsulating the concept of the time dimension. As a result, contemporary research has largely concentrated on enhancing robotic sensory, perceptual, and motor capacities, assuming short-term and nearly momentary interaction between agents (Das et al., 2015; Baraglia et al., 2016; Devin and Alami, 2016; Churamani et al., 2017). Still, human-machine confluence encompasses inherent temporal aspects that are often considered only implicitly in robotic applications, with clear negative effects regarding the integration of artificial agents into human environments. In example, robotic agents face difficulties in distinguishing between the entities involved in different past events or implement reasoning on past event sequencing, cannot feel rush or adapt to human temporal expectations and cannot effectively plan not only how, but also when tasks should be accomplished (Wilcox et al., 2012). Our recent work has addressed artificial temporal cognition, with a focus on human-like time representations and duration processing mechanisms for robots (Maniadakis et al., 2009, 2011; Maniadakis and Trahanias, 2012, 2015).

Interestingly, besides the fact that several cognitive architectures have considered for robotic systems over the last years (Langley et al., 2009; Rajan and Saffiotti, 2017; Kotseruba and Tsotsos, 2018), the notion of time is often represented rather implicitly in the knowledge base, without a clear view on the past, the present and the future of the robot life. For example, environment state changes are typically stored in a flat atemporal domain, being unable to distinguish between yesterday and a month before. The present work introduces a new cognitive framework that clearly separates between the well known notions of “past,” “present,” and “future,” which are widely adopted by humans in their daily activities.

More specifically, three important phases of human-robot interaction can be easily identified in which time has a major role. These regard (i) the representation and memorization of past experiences on a temporally rich domain to facilitate time-informed reasoning in forthcoming sessions, (ii) the perception of the temporal features of evolving real-world procedures to support action coordination with other agents in the environment, and (iii) the planning of actions to facilitate timely accomplishment of goals given the temporal constraints and the dynamic unfolding of multi-agent collaboration. Targeting the implementation of time-aware robotic cognitive systems, we have developed computational modules addressing complementary cognitive skills along the past, present and future disciplines mentioned above (Maniadakis et al., 2016b, 2017; Sigalas et al., 2017a,b; Hourdakis and Trahanias, 2018).

In this paper we present the implementation of a composite sHRI system, that comprises the aforementioned time-aware cognitive modules. The composite system (a) exploits past experiences to reason about current human needs, (b) monitors and analyzes the ongoing human activity to infer the completion time of human tasks and the user's performance profile on the task, and (c) plans synergistic robot activities properly adapted to the human profile and the progress of the task in order to accurately satisfy human expectations. The paper summarizes the integration of the time-aware cognitive modules emphasizing mostly on their interactions and the beneficial features they bring to the composite system.

To demonstrate the validity of the proposed approach, the composite system is deployed in the real world and is assessed in a complex multi-agent interaction scenario that involves two robots and a human. A series of experiments with real humans showed that complementary to the embodiment of cognitive systems (i.e., link robot actions to body characteristics), the “entiment” of robotic cognition to the temporal context of sHRI (i.e., take into account when things happened or should happen) facilitates the coordination of robot behavior with the dynamic unfolding of the sHRI scenario. Overall, the use of time-informed robotic cognition facilitates the seamless integration of artificial agents in the real world, enhancing their ability to respond more accurately, flexibly, and robustly, in full accordance to the human expectations and needs.

The rest of the paper is organized as follows. Section 2 outlines related wok on the subject of time cognition, including issues of memory, temporal predictions and time-informed planning. Section 3 outlines the proposed interaction scenario that is employed in the current work, while section 4 provides details on the implementation of the composite system and the individual components. Section 5 presents a detailed experimental evaluation of the system in a set of human-centered experiments, while section 6 concludes the paper and discusses further research directions on the subject.

## 2. Literature Review

Over the years, a number of cognitive robotic architectures have considered the implementation of high-level cognitive functions taking into account temporal information, such as the constraints on the timing of tasks (Alami et al., 1998). For example, the deliberation for the completion of multiple and diverse robot tasks can be implemented based on six types of robot functions, namely planning, acting, observing, monitoring, goal-reasoning, and learning (Ingrand and Ghallab, 2017), where the need for timing robot activities has been also considered.

The broader field of human robot interaction has been significantly facilitated by the integration of modules which provide robust solutions on well-studied problems in the field of robotics. For example, Lemaignan et al. (2017) proposed a practical implementation for social human-robot interaction combining geometric reasoning, situation assessment, knowledge acquisition, and representation of multiple agents, for human-aware task planning. Churamani et al. (2017) built a human-robot interaction module to engage personalized conversations in order to teach robots to recognize different objects. Devin and Alami (2016) developed a framework which allows robots to estimate other agents' mental states e.g., goals, plans and actions and take them into account when executing human-robot shared plans. Das et al. (2015) proposed another framework for human-robot interaction based on the level of visual focus of attention. The latter was implemented on a Robovie-R3 robotic platform in order to interact with visitors in a museum. Adam et al. (2016) implemented a framework for physical, emotional and verbal human-robot interaction on a NAO robot.

Nevertheless, in these works the temporal dimension of human-robot interaction has not been adequately considered, since the focus of the relevant implementations was on the spatial aspects of task completion. As a result, the implemented systems are unable to develop a wider conception of the timeline linking the past, the present, and the future. The present work contributes to fill this gap by proposing an integrated system that directly considers the temporal characteristics of sHRI in order to realize long-term, timely and fluent cooperation of humans and robots. Previous works related to the components of the composite system are reviewed below.

### 2.1. Knowledge Representation and Reasoning

Robotic systems that naturally interact with humans for long periods should be equipped with the ability to efficiently store and manage past memories, as well as with the ability to exploit past experiences to predict future outcomes. Still, the temporal aspects of a robotic memory system have not yet been adequately examined, with most systems using flat, non-timed memories to assimilate past experiences. Accordingly, events that occurred at different past moments can be hardly distinguished, which results in poor performance in sHRI scenarios.

A common issue when encoding past events regards the management of the stored information, given the constantly increasing storage space over time. Memory forgetting (or decay), is a biologically inspired memory mechanism (Hardt et al., 2013) which may cope with this issue. The Decay Theory (Altmann and Gray, 2000) dictates that information stored in memory tends to “fade out” and/or eventually be forgotten as time passes. Prior attempts to computationally implement memory forgetting (Ho et al., 2009; Biswas and Murray, 2015) fail to dynamically adapt to variations in task requirements and, thus, are not suitable to support long-term sHRI. A dynamic memory system is proposed by the Time-Based Resource-Sharing (TBRS) theory (Barrouillet et al., 2004), which combines decay and interference theories and, thus, allows information to be “refreshed” as well. Still, recent TBRS implementations (Oberauer and Lewandowsky, 2008, 2011), exhibit rather low memory performance in terms of recall accuracy. Moreover, Adaptive Resonance Theory networks (Carpenter and Grossberg, 1987) are also used to encode memories. However, current implementations fail to effectively model a human-inspired long-term robotic memory, either because of limitations on the perceived information (Tscherepanow et al., 2012), or on the information retrieval and refreshing (Taylor et al., 2009) or, even, because of absence of forgetting mechanisms (Leconte et al., 2016).

Evidently, the exploitation of the stored information in order to infer or predict the state(s) of the interaction would greatly facilitate sHRI (Maniadakis et al., 2007). Yet, only recently there have been some works researching memory-based inferencing. For example, Hidden Markov Models (HMMs) are used in order to infer actions consisting of a sequence of “intentions” (Kelley et al., 2008). However, the need of previously modeled and task-dependent actions, limits the employment of the system in complex real-world setups. This obstacle is alleviated in some of contemporary works, such as Nuxoll and Laird (2012) and Petit et al. (2016). The former refers to the employment of the Soar cognitive architecture in order to exploit episodic memories and enhance the cognitive capabilities of artificial systems, while, the latter, uses a-posteriori reasoning to store and manage previously acquired knowledge. However, both of these works face severe limitations regarding the constantly increasing storage requirements, negatively affecting performance in long-term HRI.

To address the aforementioned issues, we have implemented a time-aware episodic memory module (Sigalas et al., 2017a,b) for autonomous artificial agents, which enables memory storage and management, as well as sHRI state prediction and inference. As thoroughly described in section 4.1, symbolic information is stored in a temporally rich domain, which encodes the involved entities and the relation between them. Each entity is characterized by an importance factor which dictates its life-cycle and, thus, determines whether to keep or erase the related information. Separate HMMs are generated and trained on demand in order to categorize the stored information, query the memory about past events, infer “hidden” information about an episode's attributes and predict future actions.

### 2.2. Temporal Information During Action Observation

Time perception, i.e., the ability to perceive the temporal properties of an ongoing activity, is a field that remains relatively unexplored in artificial cognitive systems. This can be attributed to the fact that such investigations often require task dependent and contextual data, which are difficult to obtain. Recently, however, temporal information has been increasingly used for action recognition, which indicates that there is a strong correlation between low-level behaviors and temporal properties. For example, local spatio-temporal features (Laptev, 2005) have been showcased to have increased discriminative power (Wang et al., 2009), since strong variations in the data (such as characteristic shifts in motion and space), can be captured more accurately in the spatio-temporal domain. In this context, various descriptors and feature detectors have been proposed. In Laptev (2005), temporal information is attained by convolving a spatio-temporal function with a Gaussian kernel, while in Scovanner et al. (2007), a 3D SIFT descriptor is proposed, which extends SIFT to the time-domain. Dollàr et al. proposed the Cuboid detector, which applies Gabor filters along the temporal dimension. Temporal structure for activity recognition has also been investigated using graphical models, including spatio-temporal graphs (Lin et al., 2009) and semi-latent topic models (Wang and Mori, 2009). In contrast to the aforementioned works, which examine the temporal structure of an activity, in the current work we measure its duration. As we demonstrate the use of this information can lead to robust descriptors for the human activity.

To facilitate temporal predictions by mere observation, we have introduced Generative Time Models (GTMs) (Hourdakis and Trahanias, 2018) that can accurately predict the duration of an unfolding activity. i.e., observation models that provide in real-time estimations of temporal quantities that characterize the activity. This concept, that is predicting the time-related properties of an activity, is novel to robotics and with great potential. Information provided by the GTMs can be employed by different disciplines including human-robot interaction, scene perception, robot planning, and process modeling. In the current work, GTMs are employed to implement the observation models that allow the robot to predict the duration and completion-time of an activity performed by a human agent.

### 2.3. Time-Informed Planning of Collaborative Activities

Several works have considered the notion of time in planning solo robot behavior in the form of action sequences, frequently with the use of PDDL that uses first-order predicates to describe plan transitions (Cashmore et al., 2015), or NDDL that considers a “timeline” representation to describe sequences of temporal conditions and desired values for given state variables (Py et al., 2010), and is adopted by the EUROPA Planning Library (Rajan et al., 2007; Barreiro et al., 2012) and its subsequent advancement that considers the description of hierarchical plans (Antonelli et al., 2001). Opportunistic planning provides an alternative view for scheduling long-horizon action sequences (Cashmore et al., 2018). The use of hierarchical plans is additionally considered in Stock et al. (2015), focusing on the unification of sub-plans to improve implementation efficiency. Moreover, the high-level Timeline-based Representation Framework provides a structured library for managing operational modes and the synchronization among events (Cesta et al., 2009), or with the use of the forward search temporal planner POPF (Cashmore et al., 2014). Extensions of this framework has been used among other in industrial human-robot collaboration (Pellegrinelli et al., 2017; Umbrico et al., 2017) to ensure controllability.

To implement tasks involving multi-agent collaboration, planning algorithms often rely on constraints which provide ordering between the independently implemented activities (Morris et al., 2001; Shah et al., 2007; Smith et al., 2007; Morris, 2014). Existing approaches explore the controllability of alternative strategies, to identify plans that successfully schedule the required activities in a way that satisfies constraints until the final completion of the goal (Cimatti et al., 2016). Despite their success in coordinating pairs of interacting agents, relevant works suffer in terms of scalability because they assume a significant amount of resources to be devoted to the formulation of the full plan.

Interestingly, relevant works consider the use of time in full isolation, without the ability to blend time with other quantities for the time-inclusive multi-criteria evaluation of plans. For example, time-labeled Petri-nets have been used to accomplish fluent resource management and turn-taking in human-robot collaboration focusing mainly on dyadic teams (Chao and Thomaz, 2016). In a different work time has been sequentially combined with space to minimize annoyance among participating agents (Gombolay et al., 2013). Other works follow a similar tie-isolated formulation, representing agent interaction as a multi-criteria optimization problem. The objective function is derived from the preference values of participating agents and the temporal relations between entities are mapped on the constraints of the problem (Wilcox et al., 2013). More recent works follow basically the same formulation, representing time in the set of constraints that confine available solutions (Gombolay et al., 2017). Besides the fact that criteria such as the workload and the user preferences can be addressed with these approaches, time is largely kept separate form other quantities, thus not used for the formulation of time-informed multi-criteria objectives. Moreover, the works mentioned above do not consider predictive estimates on the performance of interacting agents and the expected release of constraints among tasks.

Recently, decentralized approaches are used for multi-robot coordination, which work on the basis of auctions. For example, Melvin et al. (2007) considers scenarios in which tasks have to be completed within a specified time window, but without allowing overlapping between time windows. Modern approaches are targeting this issue with particularly successful results in simulation environments (Nunes et al., 2012; Nunes and Gini, 2015). In other similar problems the routing of working parts is assigned to the most suitable transportation agent through an auction-based mechanism associated to a multi-objective function (Carpanzano et al., 2016). However, the relevant approaches assume auctions to proceed on an agent-centered point of view which does not consider the capacities and special skills of other team members. Therefore, it is hard to maximize the usability of all members for the benefit of the team (i.e., it might be beneficial for the team if the second optimal agent undertakes a given task).

To address the issues mentioned above, we have recently introduced the Daisy Planner (DP) (Maniadakis et al., 2016a,b), a new scheme of time-informed planning, which relies on the daisy representation of tasks and adopts time-inclusive multi-criteria ranking of alternative plans. DP operates under the assumption of pursuing immediate, locally optimal assignment of tasks to agents. This is in contrast to previous works on scheduling multi-agent interaction that typically prepare long plans of agents' activities for all future moments (Gombolay et al., 2013; Hunsberger, 2014; Cimatti et al., 2016), under the risk of frequent re-scheduling, due to external disturbances that may render current plans inapplicable. In such cases, re-scheduling may take up to a few tenths of seconds (Pellegrinelli et al., 2017). DP effectively operates as a lightweight process which minimizes the chances for re-planning in the case of unexpected events (Isaacson et al., 2019).

## 3. Multi-Agent Interaction Scenario

**Figure 1:**
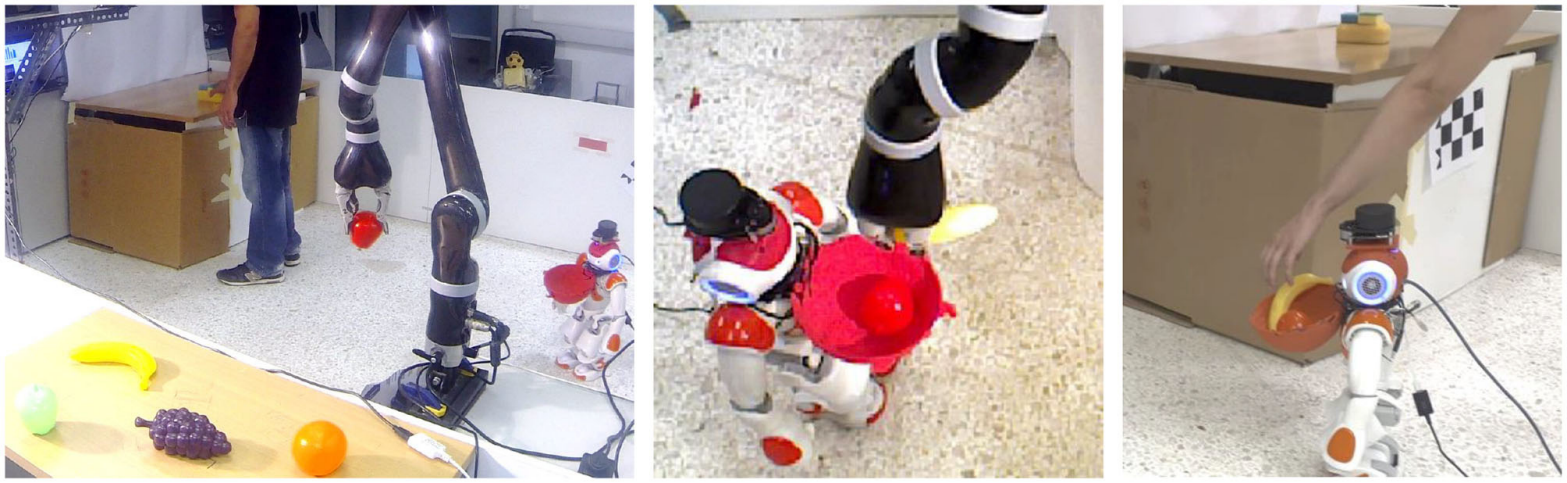
The human-robot-robot “breakfast preparation” collaborative evaluation scenario.

Without loss of generality, and for the sake of brevity, the adopted scenario considers the case of three agents: a human and two robots (a humanoid and a robotic arm). We note, however, that the proposed methodology is readily applicable to the case of more than three cooperating agents. In this section, we summarize the scenario that will be used as a motivating example for the rest of the paper (see Figure 1). The scenario assumes that the three agents cooperate for the timely delivery of breakfast to the human.

In particular, to predict the breakfast preferences of the human at a given day, the system exploits past sHRI sessions. After querying the episodic memory based on the current date (i.e., day of week, month, and season), the weather conditions and the user's mood and health, the most likely breakfast menu is inferred and forwarded to the planner that synchronizes the activities of involved agents. The collaboration scenario assumes agents to undertake the tasks they are more efficient to implement. Therefore, the human undertakes table cleaning for the breakfast to be served, given his superior performance for the task in comparison to the two robots. At the same time, the humanoid robot gets the responsibility to fetch the breakfast to the table. We use a bowl carefully mounted on humanoid's chest to support the transfer of breakfast items. This is accomplished with the help of the robotic arm, which places the appropriate number of items in the humanoid's bowl.

The number of breakfast items is dynamically determined according to the progress of table cleaning by the human. The collaboration of the two robots aims at getting and fetching the maximum number of items, after considering the performance profile of the human and additionally that the breakfast should be delivered with the minimum possible delay. As a result, a fast performing, dedicated human that is willing to finish breakfast as soon as possible may be served a minimal breakfast menu, while a very relaxed person will, on the contrary, enjoy a full breakfast menu. The above described breakfast delivery scenario facilitates the grounded assessment of the integrated time-informed system which accomplishes the fluent and timely synchronization of the robots with human activities.

### 3.1. Task Requirements and System Modules

The implementation of the above described scenario assumes the integrated performance of modules targeting diverse parts of the sHRI timeline. More specifically, to capitalize on the information gained from past sHRI sessions, the composite system must be able to (a) maintain a temporally-rich representation of past HRI events being easily searched using temporal criteria, which enables focusing on the past time periods of interest and (b) accurately infer or predict the state of the HRI, based on past experiences.

Beyond associating relevant past experience to the current situation, fluent HRI requires real-time monitoring of human activities. Our implementation achieves this (a) by developing accurate predictions on the completion of human actions with few training iterations and minimal prior information about the performed activity, and (b) by estimating human efficiency toward the real-time profiling of the interacting human.

Following the above, it is important to effectively proceed to the accomplishment of the joint goal, by coordinating the activities of team members. To this end, the multi-agent action planner is necessary to (a) maximally exploit the individual skills that each one of the heterogeneous participating agents brings into the team, and (b) implement plans that are flexibly and proactively adapted to the expectations of the user and the dynamic temporal characteristics of the collaborative scenario.

## 4. Time-Informed Multi-Agent Interaction Implementation

**Figure 2:**
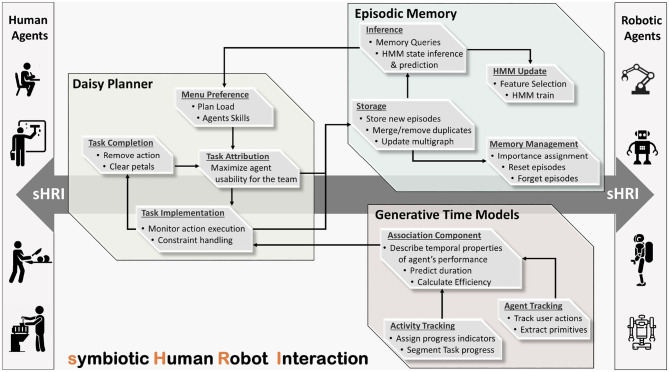
Abstract representation of the proposed sHRI system.

Figure 2 depicts the sHRI composite system, designed according to the previously delineated requirements, featuring the interconnections of the individual time-aware cognitive modules. The implemented modules are involved in distinct aspects of the sHRI process extracting complementary pieces of temporal information. As evident in Figure 2, the Episodic Memory builds on past experiences to make predictions of the human agents' needs and preferences. The relevant predictions are fed to DP which guides and coordinates agents' activities toward the implementation of the mutual goal, adequately synchronized with the evolution of real-world events. The GTM module effectively monitors the progress of human task implementation to predict remaining time and user efficiency, which are used by the DP to successfully steer and refine the cooperative plans. The extracted temporal features are additionally encoded to memory for future reference. The above described continuous interplay of Episodic Memory, GTM, and DP results into a composite system with a context and human personality driven performance that accomplishes to effectively map robot services to the needs of the individual humans.

The proposed approach implicitly addresses issues regarding the commitment of the agents to their common goal (Castelfranchi, 1995). This comes from the central coordination of the team by the planner, which eliminates motivational, instrumental, and common ground uncertainty as they are described in Michael and Pacherie (2015). In that sense, every member of the team holds normative expectation from the others, which are crucial for the successful accomplishment of the common goal (Castro et al., 2019). However besides the coordination of the team by the planner, currently, there is no means to explicitly communicate expectations or obligations among partners, an information that might be crucial for the human to understand that things are under control and the task progresses as expected. This is something that will be considered in the future versions of the system.

### 4.1. Past–Encoding of Elapsed HRI Sessions

Development of an episodic memory module, able to effectively encode past episodes on a temporally rich domain, may significantly facilitate sHRI by exploiting past experiences to infer current human needs. Specifically, the episodic memory serves to store, manage and symbolically represent user memories, in a manner which, on one hand enables storing of large numbers of entities and, on the other hand, facilitates fast and efficient search. Typically, memory stores and manages all of the perceived information. However, for the task at hand, we focus on the users' breakfast preferences, storing, and exploiting only breakfast-related entities along with their temporal (e.g., date of occurrence, duration of activity, etc.) or other (user mood and health, weather conditions, etc.) information.

Turning to the usability of episodic memory, robotic systems should ideally adapt their activities in accordance to user needs. To this end, exploitation of elapsed sHRI episodes may significantly facilitate the inference of user preferences at the given context. Valuable information stored in memory may regard the configuration of past breakfast menus (i.e., combinations of breakfast items) in association with the evolution of relevant attributes, e.g., human mood and health, on a daily basis.

#### 4.1.1. Episodic Memory Design

**Figure 3:**
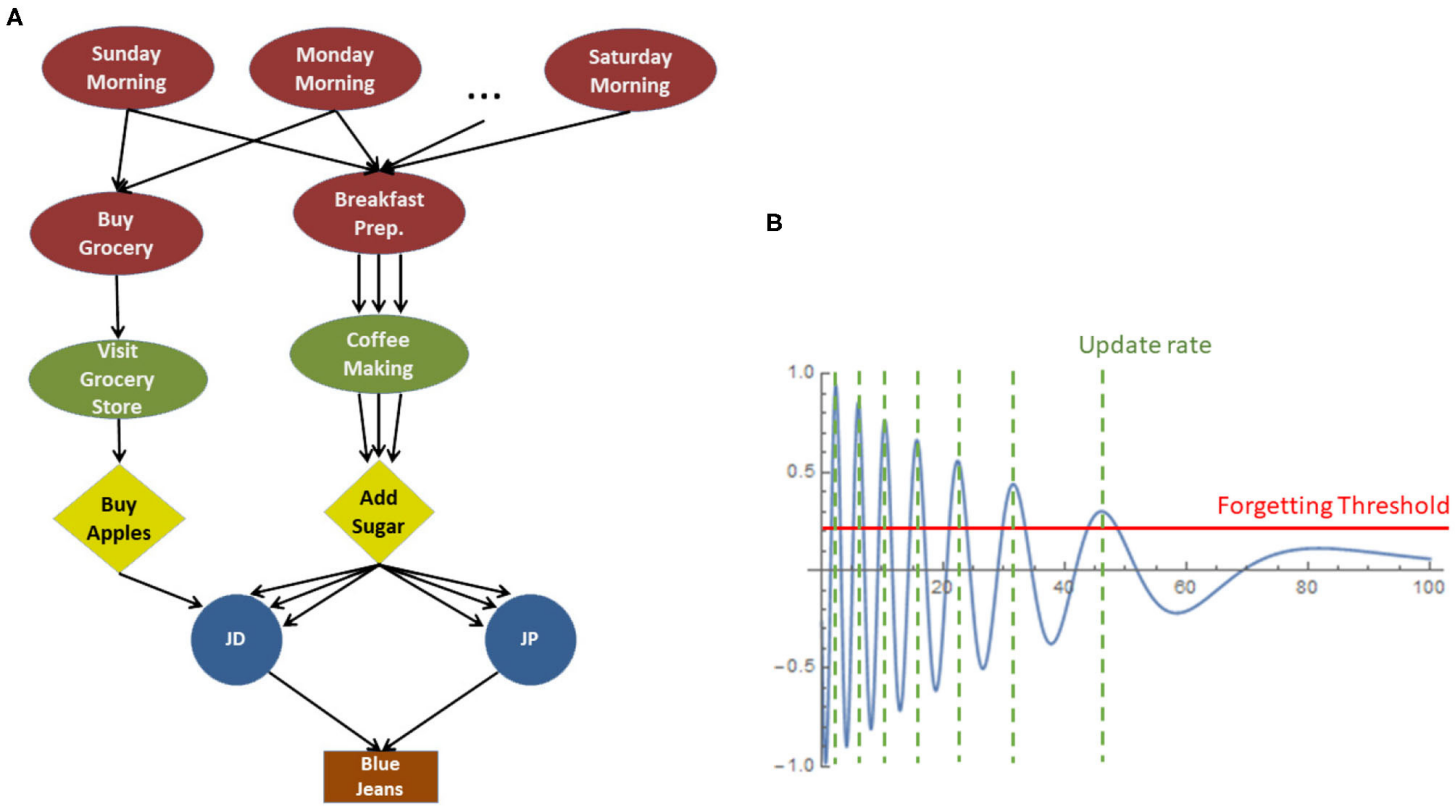
**(A)** Connected Episodic Multi-graph. **(B)** Exemplar importance function.

As described in our previous works (Sigalas et al., 2017a,b), episodes are stored in memory in the form of connected multi-graphs (Figure 3A), where the nodes represent the episode entities (i.e., Scenario, Event, Action, Actor, Object, or Feature) and the edges represent the links (bi-directional parent-child relationships) among entities, during the unfolding of the episode. Each link of the multi-graphs is assigned an importance factor which affects the entity's lifecycle and varies according to the ongoing task, i.e., entities which are more “relevant” to the task are considered more important than other “irrelevant” ones. Importance is represented by a damped sine wave (i.e., decays over time), as shown in Figure 3B. The latter is mathematically formulated as:

(1) $$\begin{array}{r} I\left(t\right)=e^{-\lambda t}\cos\left(\omega t\right). \end{array}$$

where λ is the amplitude decay constant, ω is the angular frequency and *t* represents the lifetime of the entity (*t* = 0 at the first occurrence of the said entity).

The information stored in memory is evaluated and updated periodically (at the positive peaks of the importance sinusoidal) where it can be either refreshed, merged with an overlapping entity, or forgotten, as summarized below.

- Refresh: Each time an entity is perceived or considered by the system, its importance timeline is set to *t* = 0, which indicates that it is refreshed as a memory-entity, and its importance starts decaying again following Equation (1).
- Merging: Repeated instances of the same entity (e.g., a person seen twice) are merged together. This is feasible due to the multigraph nature of the memory, allowing for multiple “incoming” and “outgoing” links, as illustrated in Figure 3A. The multigraph provides efficient memory indexing and thus facilitates fast response on memory recall queries. The given representation allows for queries of the type “*what happens every morning*” or “*what happens every time user JD is sick*,” and thus facilitates statistical analysis on human behavior and preferences.
- Forgetting: The efficient management of memory assumes a forgetting mechanism to filter out “noise,” which means to discard entities being of low importance for the stored episodes. Whenever an entity's importance drops below the so called “forgetting threshold,” the entity is deleted from the memory together with all adjacent (incoming and outgoing) association links with other entities. *Forgetting* is an iterative process, in the sense that, erasing an entity affects also its children, which, if left with no incoming links (i.e., have no other “parent” than the erased one), will be erased as well.

#### 4.1.2. Probabilistic Inference

In order to exploit the stored information and make predictions about user needs and preferences, we employ a Hidden Markov Model (HMM) inference schema (Sigalas et al., 2017a). By querying the memory, it is possible to retrieve information about past episodes, properly filtered by selection criteria. These criteria vary depending on the task at hand and the required inference; e.g., “what does JD eats for breakfast during weekdays,” or “what did JD say when the phone rang yesterday morning.”

Separate HMMs are developed on the fly—and on demand—to exploit the time-stamped data retrieved from memory. The recalled past episodes along with the selected attributes, are used to train the HMM (estimate its parameters) and infer scenario-relevant information. Training is accomplished by employing the forward-backward algorithm (Rabiner, 1989), a two-step iterative process that uses observations to predict the model state, which is subsequently used to update the model parameters. Similarly, in order to make a history-based inference of a user's preference, the HMM exploits the observed episode attributes to predict the upcoming state, based on the currently estimated model parameters. To facilitate training, a feature selection mechanism (for the current implementation we use the Boruta algorithm; Kursa et al., 2010) is periodically employed, in order to select the most relevant—to the query—features and, thus, increase inference accuracy.

HMMs are perfectly suited for the task at hand, because they provide a very flexible generalization of sequence profiles allowing for inputs of varying length. Moreover, they efficiently encapsulate the hierarchical structure of real world episodes while they are also incrementally trained, allowing for fast operation during the online scenario unfolding.

By exploiting stored information in combination with the HMM-based inference, the Episodic Memory module manages to: (a) estimate the HRI state, e.g., agent actions in relation to the objects in the scene, (b) infer hidden HRI information, e.g., user's intentions, and (c) identify abnormal unfolding, e.g., emergency events or unexpected situations. In the scenario considered in the present work, the HMM is used to predict the breakfast preferences of the user, which are further fed to the planner in order to effectively guide the scenario unfolding.

#### 4.1.3. Episodic Memory Enhancement

The Episodic Memory module was further enhanced toward the direction of increasing the performance of the HMM-based inference mechanism, in terms of both the efficiency (i.e., high inference/prediction accuracy) and effectiveness (i.e., robust and fast HMM training and inferencing). To this end, we extended the inference by: (a) not discarding the HMM after usage and (b) periodically train each HMM, instead of updating it only when queried. Initially, a feature selection mechanism [the Boruta algorithm (Kursa et al., 2010) as already mentioned above] is employed to select the most relevant -to the task- features, which are then used to incrementally train the HMM.

#### 4.1.4. Episodic Memory Key Strengths

The above presentation dictates that, in comparison to previous relevant works, the Episodic Memory module bears important features:

- Encodes episodes as symbolic information on a temporally rich domain.
- Dynamically manages (e.g., merges or forgets) the stored episode details, based on their temporally decaying importance.
- Provides accurate inference about the current or future state(s) of the HRI, based on the personalized preferences, as derived from the stored information.

#### 4.1.5. Episodic Memory Interface

The episodic memory is the representation of user's past experiences. It is directly interfaced with the Daisy Planner either for storing new information, or for inferencing the current of future state(s) of the unfolding scenario. The interface and the capacities that the memory module brings to the system, are summarized below.

**Input**. The memory accepts two types of input from DP. (a) Whenever an action is accomplished, the planner feeds memory with the relevant information; i.e., involved entities and their characteristics, general information about the current day and so forth; (b) DP sends requests about the ongoing or forthcoming states of the HRI. These requests are formulated as plain database queries, stating the predicted value(s) and the accompanying constraints.

**Output**. The output of the inference mechanism depends strongly on the incoming query. Based on a given request and constraints, the HMM is updated accordingly and the most likely response is fed to the planner.

**Role**. As evident, the episodic memory module serves two purposes: Storing and managing of past episodes, using a time-aware symbolic representation and estimating the current or future state(s) of the ongoing scenario, based on the time-stamped information and the corresponding (temporal or other) constraints.

### 4.2. Present—Temporal Features of Perceived Human Activity

Temporal information, i.e., activity duration, allows robotic systems to plan their actions ahead, and hence allocate effort and resources to tasks that are time-constrained or critical. In human cognition, such perception models are widely used (Zakay, 1989), despite the fact that our time-perception is subjective, and dependent on the implicit *sense-of-time* feeling that stems from our sensorimotor pathways (Zakay and Block, 1996). In contrast to that, robots and artificial systems may potentially perform this task more consistently, by observing and analyzing the statistical properties of the observed behaviors (Bakker et al., 2003). Recently, we have demonstrated how such duration estimates can be obtained using a model based method to derive the progress of the activity (Hourdakis and Trahanias, 2013), called Generative Time Models (GTMs) (Hourdakis and Trahanias, 2018).

#### 4.2.1. GTM Design

**Figure 4:**
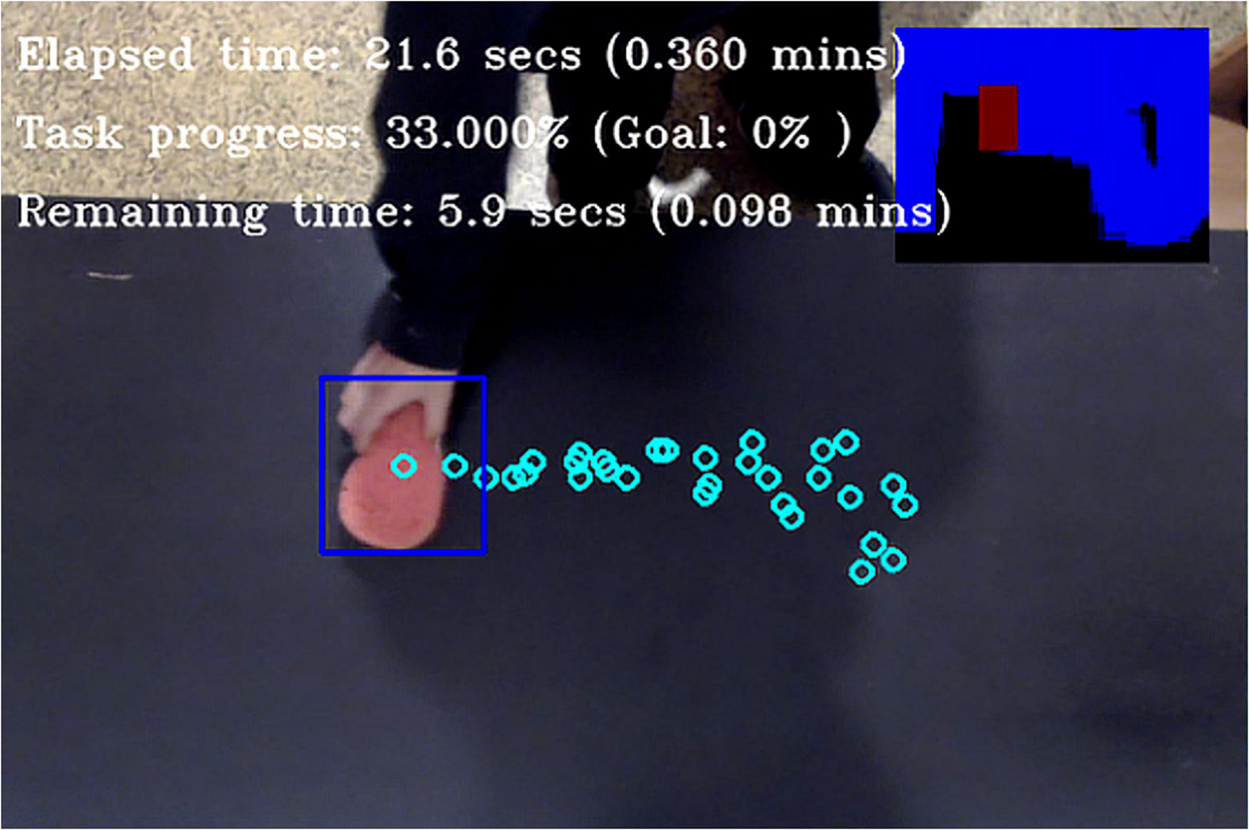
A GTM observing a human, while cleaning a table. The model extracts the low-level behavioral primitives of the human and associates them with the quantified task progress (small top-right plot). For the “wipe the table” activity, task progress is calculated by measuring the percent of the table surface that has been wiped by the sponge.

For the current implementation, GTMs are used to observe, analyze and subsequently predict the temporal properties of the human's activity (see Figure 4). This is accomplished by segmenting and decomposing the observed activity based on the human's motions. For the example of wiping the table, where we have repeating oscillatory motions, the primitives are described by their amplitude and period. To obtain the primitives, a GTM segments the signal obtained by tracking the human hand, by looking for local extrema at small Δ*t* intervals and stores their starting *t*_*s*_ and ending *t*_*e*_ times. To evaluate the local extrema it looks into the derivative of the signal, which at a point of a peak has a zero-crossing at the peak maximum.

To identify peak signal positions, the algorithm smooths the signal's first derivative, by convolving it with a Gaussian kernel, and then stores the indices of the zero-crossings on the smoothed derivative. For each index, a prominence value is calculated, which indicates whether there has been a significant change in the motion direction vector. The algorithm returns the *n* largest peaks whose prominence exceeds a certain threshold value.

The current work focuses mainly on the table wiping task, using a GTM to extract the oscillatory motions that the human produces, and associate them with the task progress (Figure 4). However, the GTM concept can easily generalize, because it uses a modular architecture, with the activity and agent components kept separate. Consequently, components from a GTM formulation can be re-used to other tasks. To create robust temporal predictors, a GTM analyzes an activity using two observation models: (1) task progress, and (2) control. The first estimates the progress of the task, i.e., how much of the activity has been completed. The latter, identifies and records information about the observed motions that appear during the activity. For each motion, it records (i) the effect it has on the task progress, i.e., how much of the task is completed each time the motion is executed, (ii) the duration of each motion, and (iii) how frequently it appears during the activity. A GTM uses this information to predict the probability of a motion occurring, i.e., how many times each motion is expected to appear in the future in the course of this activity, and how it will affect its progress.

To accomplish this, a GTM builds behavioral profiles based on motion primitives that are observed, and uses them to infer future task progression in respect to the human performance. To create its temporal predictors, the GTM employs the observed primitive models. For each primitive observed, the model segments the overall motion, and uses those segmentation intervals in order to infer how the task progresses in each interval. To make the predictions, the model follows a finite mixture approach, in which a belief is formed about the probable primitive models that will be observed by the model.

Having segmented and described each primitive that is observed, a GTM approximates the activity progress *O* in future time-steps, using a finite mixture model. To estimate *O* we sum the expected progress to the task by each observed primitive, weighted by the primitive's probability, as shown in Equation (2):

(2) $$\begin{array}{r} O\left(t\right)=\overset{k}{\underset{i=1}{\sum }}p\left(i\right)\phi_{i} \end{array}$$

where *p*(*i*) are the weight factors, that satisfy *p*(*i*) ≥ 0, for all integers *i* ∈ 1, *k*, and $\overset{k}{\underset{i=1}{\sum }}p\left(i\right)=1$, while $\phi_{i}=\int_{0}^{t}f_{M_{\left(i\right)}}\left(t\right)dt$ provides the overall contribution of the primitive *i* to the task progress, with *f*_*M*_(*i*)__(*t*) being the function that describes how each primitive contributes to the task progress at a certain point in time. A GTM uses Equation (2) to predict future states for the activity progress, i.e., the expected change for the task progress is calculated using the probability of observing the primitive, and how much the latter contributes for the task completion. Using Equation (2), one can derive useful information about the observed activity. Given the weight factor *p*(*i*), ∀*i* ∈ 1, *k* for all primitives one can estimate, using Equation (2), how the task progress will change from *t* to $t_{k}=\overset{k}{\underset{i=1}{\sum }}\left(p\left(i\right)d_{i}\right)$:

(3) $$\begin{array}{r} O\left(t+t_{k}\right)=O\left(t\right)+\overset{k}{\underset{i=1}{\sum }}\left(p\left(i\right)\int_{t}^{t+d_{i}}f_{M_{\left(i\right)}}\left(t\right)dt\right) \end{array}$$

Equation (3) provides an estimate of the activity progress forward in time, using the *f*_*M*_(*i*)__ as basis functions. Based on Equation (3), robust predictions on the duration of an activity can be obtained. For the current implementation, the model is used to provide estimates that can infer how long a human agent will require in order to finish the table wiping task. A more detailed presentation of the above model can be found in Hourdakis and Trahanias (2018).

#### 4.2.2. GTM Enhancement

For the current implementation, GTMs were extended to estimate the efficiency of the agents when performing a task, i.e., the extent to which the actions performed are productive toward finishing the activity. To this end, efficiency is relevant to self-learning, and measures the quality of task execution for a given activity. To accomplish this, we measure for each primitive the fraction of the percent of the activity it completes against its duration (Equation 4).

(4) $$\begin{array}{r} e_{h}=\frac{Pr_{t}}{Pr_{d}} \end{array}$$

where *Pr*_*t*_ indicates the percent of the task that has progressed due to a primitive, and *Pr*_*d*_ the duration of that primitive. Both quantities are readily available and computed using the GTM mathematics, as described in Hourdakis and Trahanias (2018). Efficiency values are estimated online while the human activity progress, and they are sent to the planner for further processing and timely adaptation of the multi-agent collaboration strategy.

#### 4.2.3. Key Strengths

GTMs can make accurate predictions online, making them an ideal candidate to process the immediate planning context of an interaction session. Their key strengths are summarized below:

- Provide robust predictions with few training iterations.
- Use a modular architecture, with segregated Control and Activity observation components, which allows the concept of GTMs to generalize across tasks.
- Can extract additional metrics, such as efficiency, which are useful for planning.

#### 4.2.4. GTM Interface

GTMs provide a module that profiles and predicts the future performance indicators of a human. Below we outline the module's input, output conventions and role in the composite system.

**Input**. Input in the GTM is in the form of visual images, obtained by a camera. For the current experiment, the camera is mounted approximately 2 m above the table, in order to observe the table wiping task. At initialization, the human marks the rectangle containing its hand, which is used by the GTM for tracking.

**Output**. Using the raw camera images and tracker input, the GTM identifies the primitives of the human, and estimates two measures: (1) the expected duration of the experiment, and (2) the human's efficiency. This information is subsequently sent to the planner.

**Role**. The role of the GTM is to extract and estimate a temporal profile of the human participating in an interaction session. This profile is used to predict future task states, and temporal parameters regarding the human's performance.

### 4.3. Future—Plan Robot Behaviors in Coordination With Human Activities

The fluent coordination of multi-agent activities plays a crucial role in the joint accomplishment of goals. We have recently introduced (Maniadakis et al., 2016a,b), a time-informed planner that attributes tasks to agents in a step-by-step manner, accomplishing the effective coordination of multiple agents (see also, Isaacson et al., 2019). The planner assumes the daisy-like representation of the composite behavior and is thus termed *Daisy Planner* (DP). In particular, each task consisting of an action sequence is represented as a petal of the composite daisy graph. Constraints link actions among tasks that can be implemented in parallel, to indicate that the completion of a certain action is a prerequisite for the action of the other task to commence.

The planer is designed as a lightweight immediate optimal planning module, particularly appropriate for dynamic multi-agent environments where unexpected events (e.g., a phone ring, or the drop of human performance) may increase the implementation time of tasks and trim off team productivity. The DP avoids searching extended solutions of complex agent-task assignments that span over the future timeline, in order to flexibly and with low-cost adapt to unpredicted circumstances. The local view of the planner makes processing particularly light-weight, because it does not synthesize and does not compare complex future scenarios as it is the case with previous works (Wilcox et al., 2013; Gombolay et al., 2017), which additionally suffer from the need of resource-expensive rescheduling when unexpected events occur.

The planner functions under the assumption of task assignment to agents based on their availability. In order to find the best petal fit for a given non-busy agent, DP considers the capacities of all team members and builds upon the skills that the current agent brings into the team, trying to make it maximally useful for the team and the given interactive scenario. This is different to existing approaches based on Timed Petri Nets (Chao and Thomaz, 2016) in which agents are pre-assigned the sets of actions they are implementing. The planner effectively combines time with other quantitative measures that outline key features of task implementation, such as efficiency, robustness, even fatigue, and like/dislike for the case of humans, in order to construct composite time-inclusive criteria for ranking alternative multi-agent plans. This is in contrast to other works that include time as a constraint that confines the search of viable solutions (Gombolay et al., 2017).

#### 4.3.1. DP Design

The setup of the DP assumes the identification of tasks that have to be fully implemented by a single agent. For example, to implement the task “pour oil in salad,” the very same agent must grasp the oil bottle, move it above the salad, pour the oil and put the bottle back on the table. Therefore, “pour oil in salad” is represented as a petal of the composite daisy-represented scenario. Each task/petal consists of a sequence of actions that start and end at the rest state.

To initialize DP, the duration and quality of implementation for all possible action-agent pairs is provided to the planner. Duration information is obtained by summarizing previous trials and has the form of (min, max) experienced time. The quality of implementation is set by the experimenter, e.g., the humanoid is declared with poor quality to grasp and manipulate complex objects, but high quality to navigate. Using this information, the DP successfully matches tasks with the skills of individual agents, being able to construct particularly productive teams, which may flexibly consist of heterogeneous agents.

The planner employs the fuzzy number representation of time to facilitate the processing of temporal information (Maniadakis and Surmann, 1999). Following the well-known representation of fuzzy numbers in trapezoidal form with the quadruplet (*p, m, n, q*), a fuzzy duration in the form “approximately *a* to *b* moments” is represented with the fuzzy trapezoidal number (0.9*a, a, b*, 1.1*b*). In the current work, parameters *a* and *b* correspond to the minimum and maximum experienced implementation times, as discussed above. The use of fuzzy calculus (Dubois and Prade, 1988) provides the means to effectively associate the temporal properties of individual actions, predict delays of alternative planning scenarios and enable corrective measures to be taken in order to enforce the coordination of the individual activities (Maniadakis and Trahanias, 2016a). Moreover, it facilitates the comparison of agents' utility on different tasks (by combining implementation time and effectiveness), therefore enabling the use of optimization criteria for the locally optimal attribution of tasks to agents. In particular, each non-busy agent is assigned a new task in a way that maximizes agent's utility for the team, given the current, short-term view of team performance. Full implementation and assessment details of DP have been presented in Maniadakis et al. (2016a,b), and are not repeated here and re not listed here for the clarity of presentation.

In short, the immediate optimal planning approach followed by the DP, aims at naturalistic, smooth and low anxiety collaboration among the participants rather than generating globally-optimized minimum-time behaviors. This is particularly the case in most human daily collaborative tasks where participants share jobs based on expertise, tiredness, etc.

#### 4.3.2. DP Enhancement

The current article elaborates on the management of constraints which prioritize action execution between tasks that may implement in parallel but constrain each other. In particular, the present work considers the time each constraint is expected to release in order to make more informative decisions when attributing agents to tasks.

**Figure 5:**
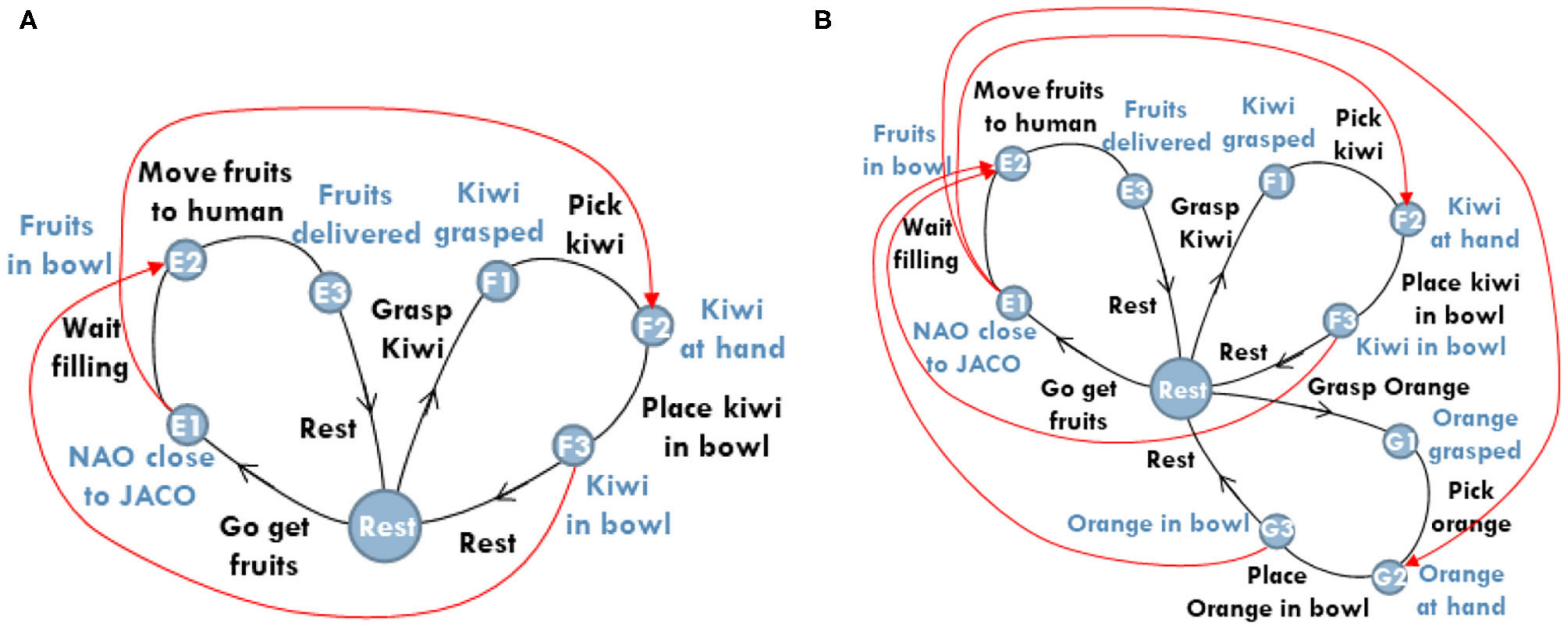
Exemplar cases of plan constraints, depicted in red. **(A)** Illustrates the case of two constraints used to prioritize the actions of petals implemented in parallel. **(B)** Illustrates the case of two constraints on *E*2. As soon as *F*3 is reached, the planner releases the constraint *G*3 → *E*2, to comply with temporal constraints (i.e., avoid the delay of breakfast delivery).

For example, consider the case shown Figure 5A, where two constraints (shown in red) determine the ordering of action execution between agents working on different tasks. The first constraint (top red arrow) specifies that the humanoid robot must have completed its way to the robotic arm, before the latter starts placing the fruits in the humanoid's bowl. The second constraint (bottom red arrow) specifies that the fruit should be in the bowl before the humanoid departs to deliver breakfast to the human.

To effectively manage time resources, the planner needs to know when the humanoid is expected to arrive close to the robotic arm. The planner knows that navigation was initiated, for example, 34 s ago and the whole navigation takes approximately 50–60 s, represented by the fuzzy number quadruplet (45, 50, 60, 66). Therefore, the remaining time for humanoid's navigation is *t*_*hr,n*_ = (45, 50, 60, 66) − 34 = (11, 16, 26, 32). At the same time, the time needed by the robotic arm for grasping the fruits is known from previous trials to be *t*_*ar,g*_ = (10.8, 12, 20, 22) and for picking the fruits *t*_*ar,p*_ = (2.7, 3, 4, 4.4). Thus, the total time needed by the arm to prepare fruit placement is *t*_*ar,g*+*p*_ = (13.5, 15, 24, 26.4). The difference^1^ between *t*_*hr,n*_ and *t*_*ar,g*+*p*_ according to the LR-calculus, results to the fuzzy number (−15.4, −8, 11, 18.5) which reflects the predicted desynchronization of the two agents. The defuzzification of this interval (implemented by the classic graded mean integration representation; Khadar et al., 2013) results into 1.516 s, indicating that robotic arm is not yet delayed, but should soon proactively initiate fruit grasping to avoid introducing idle time in humanoid's schedule. It is noted that the forward looking, proactive release of constraints based on the real-time monitoring of scenario unfolding is a new feature that has not been addressed by previous works.

Moreover, in the current work DP is enhanced to develop personalized sHRI that exploits real-time human temporal profiling, thus introducing an additional new feature to the state of the art. To slightly complicate the scenario considered in the present study, we assume that a high performing human might probably be in a high arousal state, he/she highly dislike delays, and would only be satisfied with the delivery of the breakfast immediately after table cleaning. In contrast, a low performing person may be in a low arousal state and most likely would thoughtlessly accept small delays in the order of a few seconds, with the benefit of having more fruits delivered. The above builds on the well studied link of emotional state and time perception, which shortly claims that time seems to fly when we are in a high arousal state, and to drag on when we are bored (Droit-Volet and Meck, 2007).

Following the scenario, human efficiency *e*_*h*_ ∈ [0, 1], defined in Equation (4), is an important parameter for determining the number of breakfast items to be served to the human. To explain this further, we consider a second example focused on multi-agent collaboration (see Figure 5B). We assume that the planner is informed of the estimated remaining time for the human to complete the cleaning task *t*_*h,cl*_ and his current level of efficiency *e*_*h*_. The robotic arm has just added an item in humanoid's bowl and the planner is ready to decide whether there is enough time for the arm to add one more item in the bowl, or, the humanoid should start navigation toward the human, to avoid delay. The planner knows by experience that the total time required by the arm to grasp, pick and place an item in humanoid's bowl is *t*_*ar,g*+*p*+*p*_ and additionally that the time needed by the humanoid to deliver breakfast to the human is *t*_*hr,d*_. The sign of the defuzzified difference between the total robot synergy time and the human time scaled by his/her efficiency is used to decide task allocation as described below:

**Table:**
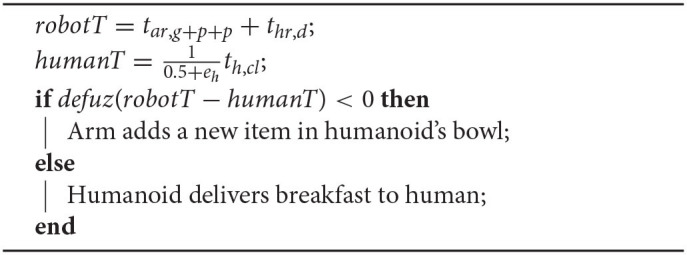


Clearly *t*_*h,cl*_ and *e*_*h*_ can drastically affect planner decisions. This is not only because decision making assimilates the latest estimate of human completion time *t*_*h,cl*_, but additionally because efficiency values *e*_*h*_ are used to scale the human available time. In particular, *e*_*h*_ values close to one reduce further the human available time *humanT*, to stress the assumption that a highly efficient human in high arousal does not accept delay in breakfast delivery. On the other hand, *e*_*h*_ values close to zero have an opposite effect increasing the available time *humanT*, thus indicating that relaxed humans would tolerate a short delay under the benefit of having a richer breakfast.

Noticeably, besides the fact that previous works have also considered user efficiency in multi-agent interaction (Gombolay et al., 2017), the real-time assimilation of the relevant information to adapt team performance is an aspect that has not been addressed so far by existing works, but is greatly and inherently facilitated by the immediate planning approaches adopted by the DP.

It is noted that even if the planner decides the immediate depart of the humanoid, it may often be the case that active constraints inhibit the humanoid's navigation. This might be because not all three items are yet placed in the humanoid's bowl. This is particularly the case depicted in Figure 5B. However, given that the humanoid should preferably depart to avoid delay, the planner has the option to release all the constraints inhibiting the humanoid's departure, making the robot free and ready to go.

#### 4.3.3. Key Strengths

In comparison to previous relevant works, the Daisy Planner:

- Implements plans that are flexibly and directly adapted to the dynamic unfolding of the collaborative scenario, which due to the immediate planning approach adopted, avoids re-planning of multi-agent activities.
- Operates as a lightweight process that effectively scales to handle large multi-agent teams, because the complexity of short-term task attribution increases linearly with the number of agents.
- Exploits the predicted temporal features derived from the real-time monitoring of agents' activities in order to enhance coordination between team members and more accurately meet the expectations of users.

#### 4.3.4. DP Interface

The planner is the eye to the future for the composite system. It is directly interfaced with the Episodic Memory and GTM to develop and maintain a dynamic third-person perspective on user expectations. The interface and the capacities that the planner brings to the system, are summarized below.

**Input**. The planner sends queries to the memory to get back inferred estimates of the human preferences which are interpreted as the goal that the composite team has to achieve. To adequately orchestrate interaction, the planner is informed about the progress of action execution by the individual agents and the efficiency of human on the action he/she is currently implementing.

**Output**. The planner tracks the implementation of tasks by the individual agents and requests the timely execution of the relevant actions to enhance coordination. Additionally, it informs the memory about the evolution and the implementation details of the composite scenario (which agent implemented each task, when the implementation started and how long it took), which are stored for future reference.

**Role**. The DP actively guides the participating robots to map their activities on human expectations and times. It is implemented as a lightweight procedure that (i) composes effective multi-agent teams consisting of heterogeneous members, (ii) exploits information on the human behavioral profile to develop assumptions about his/her temporal expectations and accordingly adapt the performance of robotic agents, (ii) enforces the timely interaction among agents considering the inter-dependencies between the individual activities, (iii) provides to the system a third person perspective on how humans perceive the notion of time, and (iv) flexibly adapts robot activities to the performance of the other team members (e.g., robots may speed up to catch up a fast performing human).

## 5. Experimental Results

The proposed approach has been implemented and validated in a realistic scenario that regards the interaction of three agents, i.e., one human and two robots, as summarized in section 3. In the current work two robots are used, namely the Kinova JACO six-joint robotic arm manipulator and the Softbank Robotics NAO humanoid, which contribute to the robot team complementary skills for serving the human. The details of technical implementations and the experimental setup are described below, followed by the real-world and the quantitative assessment of specific modules and the composite system as a whole.

### 5.1. Enabling Robotic Skills

To implement the scenario discussed throughout the paper in the real world, a variety of robotic modules have been implemented to facilitate task accomplishment by the individual agents and guarantee the success of the synergistic multi-agent performance.

#### 5.1.1. NAO Mapping and Localization

Initially, a 2D-map of the environment is created utilizing a planar-LIDAR mounted on the NAO robot's head and the leg odometry with the ROS mapping package^2^. This map is a typical occupancy grid highlighting where obstacles are located. Subsequently, the robot can localize itself in the map with a particle filter fusing in real-time the laser scan readings and the leg odometry. This is done with the Adaptive Monte Carlo Localization ROS package^3^.

#### 5.1.2. NAO Path and Step Planning

Having defined a goal where the robot should navigate to, a plan is generated with the move_base ROS package^4^. First, a global planner based on the Dijkstra algorithm is employed to search for an optimal, obstacle free trajectory. This trajectory is fed to a local planner, in our case the Timed-Elastic-Band (TEB) planner (Rösmann et al., 2013) to compute the motion-parameters which are necessary for the robot to follow the prescribed trajectory. This local planner directly considers obstacles that can unexpectedly appear (i.e., someone passing in front of the robot) and the robot's kinematic constraints. The obtained desired velocities are then transformed to desired footstep locations with our custom ROS humanoid robot step planner.

#### 5.1.3. NAO Walk Engine

Subsequently, the desired step locations are fed to the walking engine that computes in real-time the walking pattern (Piperakis et al., 2014) and tracks that pattern using onboard proprioceptive sensing such as the IMU, joint encoder, and pressure measurements (Piperakis and Trahanias, 2016; Piperakis et al., 2018) and/or external odometry measurements (Piperakis et al., 2019a,b) along with the current contact status (Piperakis et al., 2019c), to achieve fast and dynamically stable locomotion. The latter is vital to the success of the task since the humanoid carries a significant weight (mounted LIDAR and bowl with items) and still manages stable omnidirectional walk. The same module is also responsible for maintaining NAO's balance during fruit filling.

#### 5.1.4. Jaco Motion Planning

For the Jaco arm, safe and accurate pick and place actions for the end-effector are learned through an offline imitation process as proposed in Koskinopoulou and Trahanias (2016) and Koskinopoulou et al. (2016). Those actions are executed via inverse kinematics in order to pick all requested breakfast items and place them in the bowl carried by the NAO robot.

#### 5.1.5. Jaco Object Detection

The actions are triggered by visual detection of the corresponding items with an RGBD camera based on their color information with the cmvision_3d ROS package^5^. First, a detected utensil is picked by JACO and afterwards is placed when the bowl is detected.

### 5.2. Experimental Setup

To examine the performance of the system in the real-world, twenty volunteer FORTH employees have been recruited to interact with the robots, following the scenario summarized in section 3. In particular, the cohort for the sHRI study consisted of 14 men and 6 women with an average age of 34.5 ± 4.6 years (range, 27–45 years).

Significant variations have been observed in the times spend by the users to implement the table cleaning task. In this context, the time spent by a user is directly correlated to his/her efficiency for the task (see Equation 4) estimated for each participant. Table 1 summarizes task completion times per participating agent for each run with a different user. Clearly, in all cases the NAO-JACO pair has accomplished to successfully and timely deliver the fruits menu to the human. In most cases the robots complete their tasks prior to the human, as evidenced by the comparison of the last and third-to-last columns.

**Table 1:** Real-User experiment data.

	**NAO to JACO**		**Place 1st fruit**		**Place 2nd fruit**		**Place3rd fruit**		**NAO to human**		**Table cleaning**	
**User**	**Start**	**End**	**Start**	**End**	**Start**	**End**	**Start**	**End**	**Start**	**End**	**Start**	**End**
U1	1	55	1	66	67	108	109	150	152	190	1	185
U2	1	52	1	64	65	100	101	139	141	183	1	182
U3	1	52	1	65	66	105	106	141	143	182	1	191
U4	1	51	1	63	64	101	102	139	141	181	1	188
U5	1	57	1	74	75	111	112	152	154	192	1	194
U6	1	54	1	71	72	107	108	153	155	190	1	178
U7	1	55	1	66	67	105	–	–	107	145	1	155
U8	1	52	1	64	65	109	–	–	110	152	1	146
U9	1	48	1	59	60	104	–	–	105	142	1	168
U10	1	49	1	64	65	102	–	–	104	144	1	171
U11	1	51	1	67	68	107	–	–	108	149	1	147
U12	1	55	1	68	69	108	–	–	110	150	1	163
U13	1	47	1	61	62	101	–	–	103	142	1	161
U15	2	53	2	68	69	112	–	–	113	153	1	157
U15	1	53	1	65	66	110	–	–	111	155	1	152
U16	1	55	1	65	–	–	–	–	67	115	1	121
U17	2	54	2	62	–	–	–	–	65	117	1	134
U18	1	51	1	61	–	–	–	–	63	112	1	129
U19	1	53	1	64	–	–	–	–	67	118	1	123
U20	1	48	1	60	–	–	–	–	63	110	1	127

**Figure 6:**
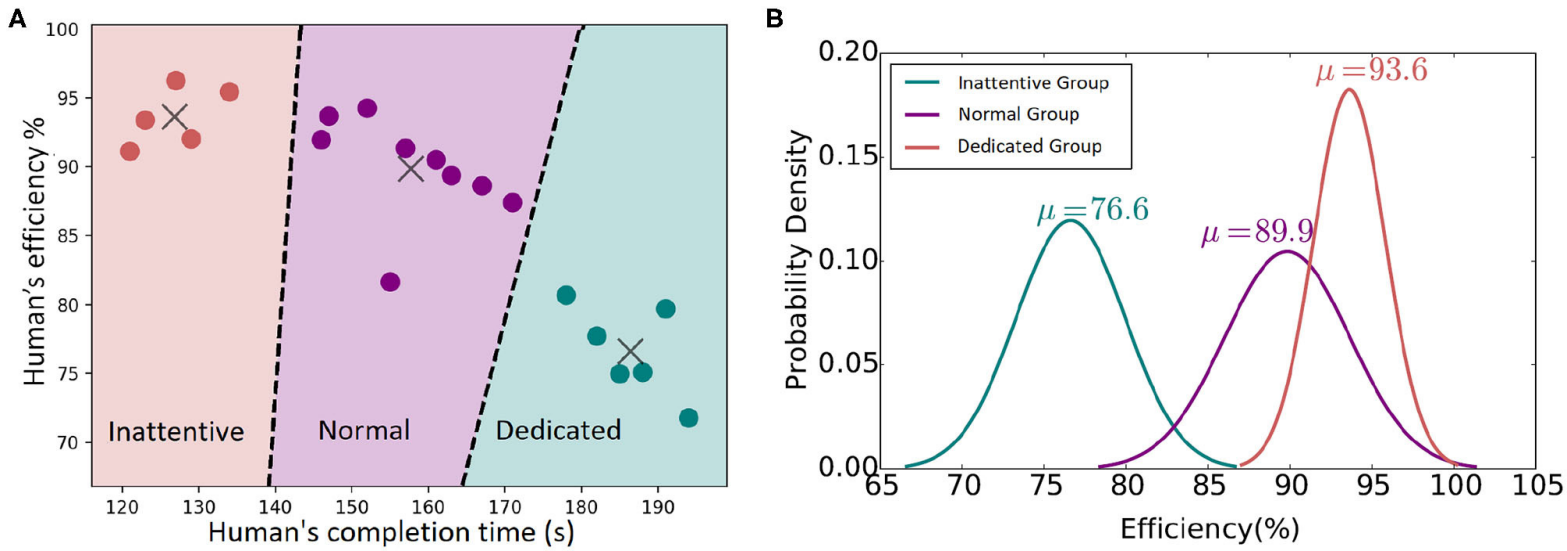
**(A)** Human's behavior classification. **(B)** Normal distributions of each group's efficiency.

To classify the experiment participants based on their performance, we use a k-means clustering approach to identify participants with similar behavioral characteristics. In particular, for each participant, the table cleaning time and their average efficiency are provided as input to the k-means algorithm. Multiple clustering arrangements have been explored, assuming two, three, four, and five clusters. The three-clusters arrangement is observed to group human behavioral features with sufficiently low classification cost (see Figure 6A). The efficiency distribution of each group is additionally depicted in Figure 6B, along with the relevant means.

The three clusters are assigned the labels Inattentive, Normal, and Dedicated as an implicit but representative description of the different human behavioral profiles observed. The grouping of human participants into Inattentive, Normal, and Dedicated is reflected in Table 1 presentation and has been further adopted in the present work as a means to provide a more informative analysis on the evaluation of the system in human-robot interaction as presented below in section 5.3.

In addition to real-user experimentation and in order to explore the performance of the composite system in a broad range of situations and user profiles, a simulation environment has been implemented, which facilitates rigorous quantitative assessment of the proposed time-aware sHRI approach. To adequately assess the flexibility of the proposed solution, we simulate human agents assuming the same three types of user profiles, namely, Inattentive, Normal, and Dedicated, as they have been observed in the real-world experiments. The details of the underlying experimental procedure are given in section 5.4.

### 5.3. Real User Evaluation

#### 5.3.1. Memory-Based Inference

The scenario assumes the inference of the breakfast preferences of the human, based on past experiences. To this end, the system capitalizes on the probabilistic inference capacity of the episodic memory module to predict the user's breakfast choice, after considering the menu combinations he/she had in the past. The foreseen breakfast menu is fed to the planner which guides the two robots in fetching breakfast items and delivering them to the collaborating human, at the right time.

A data collection procedure has been adopted to provide the ground truth for assessing the performance of the episodic memory inferencing. In particular, we asked the 20 participants to provide their breakfast preferences, i.e., a selection of three fruits among six available fruit options (20 possible triplets), for 35 consecutive days. Additional information was also provided, i.e., current date, weather conditions, scenario location, user's clothing, state of arousal, fatigue, health, and mood, summing up to 8 attributes. We divided the dataset into two parts: the first 25 days are stored in memory as past experiences in the form of multi-graph episodes, while the last 10 serve as the test set for system predictions.

The data considered as “past experiences” are used for training the HMM inference engine. Specifically, in order to infer the user preferences for the *ith* day (*i* > 25) the memory is queried to obtain insight on the relevant breakfast menus the user had in the previous 1, …, *i* − 1 days. This information is used to train an HMM, which is employed to predict user's breakfast choices on the *ith* day. The actual user choice at the given day is used as ground truth for assessing the success of breakfast predictions.

**Figure 7:**
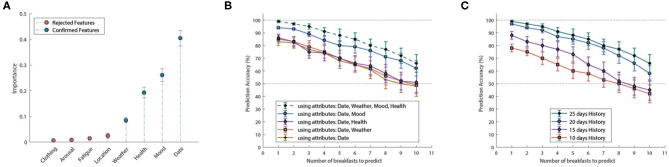
**(A)** Feature importance according to Boruta feature selection algorithm. Features with red color are characterized as non-important and are rejected, whereas features with green color are characterized as important and are accepted. Inference accuracy w.r.t. prediction days and **(B)** number of attributes, **(C)** training period.

The inferencing mechanism has been evaluated against multiple configuration setups. In particular, we assessed the effectiveness of the HMM-based inferencing by making predictions of the users' breakfast menu preferences for a period of 1–10 days ahead (i.e., days 26–35). For performance enhancement we used only those attributes which are statistically significant to the system. In particular, Figure 7A illustrates the importance of the observed features, as computed by the Boruta feature selection algorithm. Important features, denoted with green color, are accepted to be used for state inferencing, whereas non-important features, illustrated with red color, are rejected.

In order to assess the performance of the inference mechanism, we conducted two sets of experiments with (i) varying input configurations of the four important attributes, namely date, weather conditions and user's mood and health, and with (ii) varying HMM training configurations using the most recent 10, 15, 20, or 25 days of training “history” (Figures 7B,C, respectively). In the first case, the HMM has been trained using 25 days of “history” and the corresponding combination of attributes, while in the second case the whole set of important attributes has been used for training, along with the corresponding “history.”

Regarding the first set of experiments, Figure 7B demonstrates how the different attributes, i.e., the HMM observations, affect system performance. As observed, not all attributes have the same effect on the accuracy of the inference mechanism, as also implied by the relevant importance. For example “Weather Conditions” and “User's Health” play a minimal role, in contrast to the “User's Mood” which significantly improved inference performance. On the other hand, Figure 7C highlights the impact of the training period, i.e., number of past days (“history”) used for the HMM training, on the inference accuracy. Clearly, performance is improved as the number of past days included in the training increases, i.e., more information is provided to the HMM.

In short, the proposed inference mechanism, has made highly accurate breakfast menu predictions, compatible with the personalized preferences of the individual users. Naturally, prediction accuracy decays as the looking ahead period, i.e., the period for which the mechanism is required to make predictions, extends to the future. Nevertheless, inference accuracy remained above 90% for the first 4 days, while managing to provide with adequately accurate prediction (above 80%) for a period of up to 7 days.

#### 5.3.2. Duration Prediction

GTMs are used to predict the duration for the table cleaning task implemented by a human. For this reason, we deploy a GTM that is able to estimate accurately the time required for a human to finish the task. The experimental setup consists of a room, containing a table. A logitech HD camera is mounted at 2 m above the floor, in order to have visibility of the whole surface.

To detect the progress for the wiping the table activity, we determine a table region that designates the area to be wiped. In addition, the task observation module employs the output of the tracking module (i.e., location of the sponge), in order to identify what percent of the extracted plane has been wiped. To accomplish this, we use a hitmap as a matrix with dimensions equal to the table's width and length. As the sponge wipes the table, the matrix cells, whose rows and columns correspond to the {*x, y*} coordinates of the sponge's position, are being updated from 0 to b (a scalar value), in order to indicate that the surface in these coordinates has been wiped. The value b ranges from [0 to 10] indicating the strength of the sponge while cleaning. In our experiments, b is set to 6 to match the used sponge strength. The sum of the matrix cells, divided by the product of the matrix's rows, columns and value b, provides the percentage of the surface being wiped. The value b is used to reflect the fact that when wiping a surface, one usually wipes the same area more than once. Therefore, while wiping -and updating the table matrix- one should only consider a region of the table clean if it has been wiped over b times.

To analyze the human activity we observe the wiping motions by tracking the center of the sponge, using the color based tracking framework proposed in Henriques et al. (2012). We then calculate the motion vector changes in each wiping segment, and use them to identify new primitive movements. These primitives are labeled according to the effect they have on the task progress, and used as predictors for the activity. Hence, having obtained the task progress, GTMs employ information from the observed primitives to detect the intervals that correspond to each primitive. Frequently occurring intervals within the activity, are used as predictors for the task progress. In Table 2, we illustrate the averaged results obtained from the three different user groups.

**Table 2:** Average duration estimates, ground truth duration, and error measured during the wipe the table experiment, for the Inattentive, Normal and Dedicated user profiles.

**User profile**	**Av. Predicted time**	**Av. Gr. truth**	**Error [sec]**
Inattentive	177.3	186.2	8.9
Normal	149.2	143.8	5.4
Dedicated	136.9	128.6	8.3

As can be verified by the obtained results, duration predictions are robust since they fall below 10% of the overall activity duration for all three user groups. Therefore, they can support the implemented scenarios.

#### 5.3.3. Evaluation of DP-Driven sHRI

**Figure 8:**
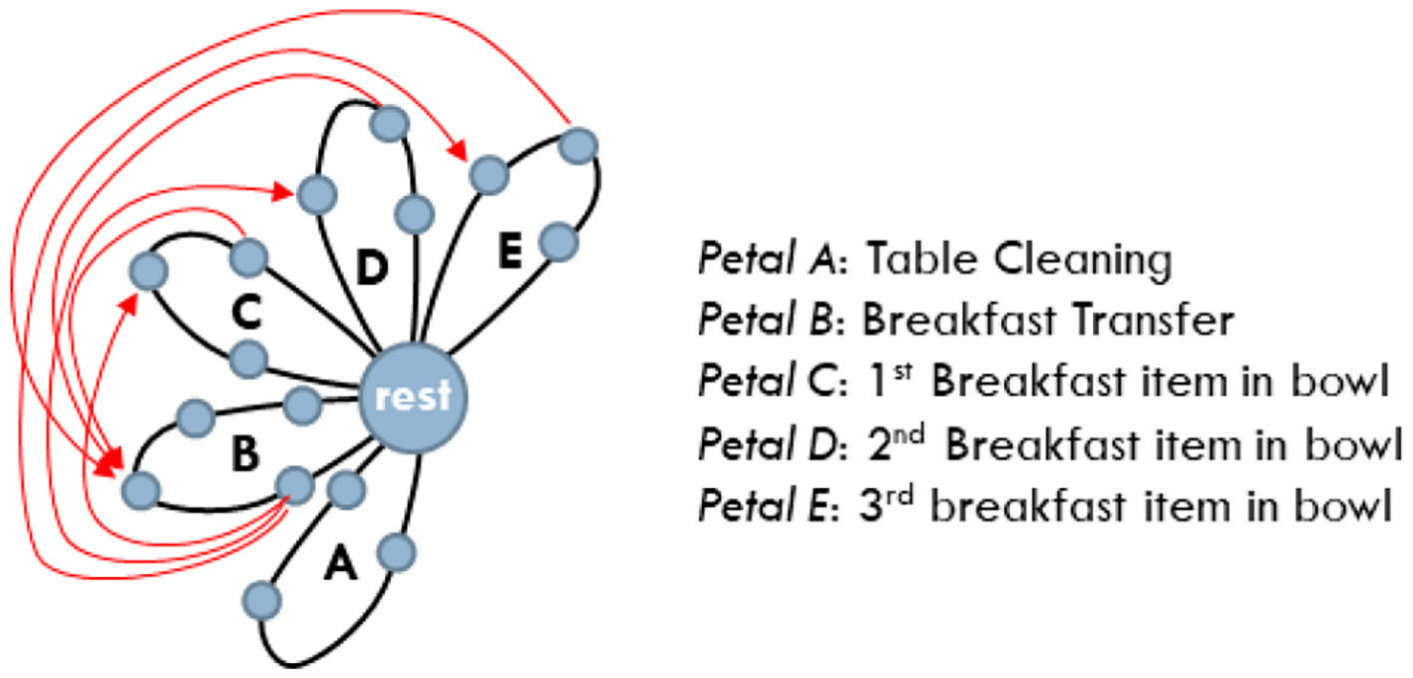
The daisy representation of the tasks involved in the timely breakfast delivery scenario. Constraints among actions are depicted in red.

The current section focuses on the evaluation of the Daisy Planner module, used to coordinate the activities of the agents involved in the timely breakfast delivery scenario. The behavior to be implemented by the three agents is separated into five tasks represented by five distinct petals on a Daisy Plan, as shown in Figure 8. The tasks are further split into actions as tabulated in Table 3. For all three agents, the same table shows the (min, max) times of action execution -as previously mentioned in section 4.3—and the corresponding efficacy level represented by the numbers 1 (lowest), 3, 5, 7, 9 (highest). Efficacy values are defined by the experimenter, prior to the actual experimentation. The manual setup of the planner rises some scalability issues when addressing incrementally more complex collaborative problems, since the skills of the individual agents and how they fit to the domain tasks have to be explicitly defined. Still, this is largely unavoidable and to the best of our knowledge, there is no multi-agent collaboration method that assumes minor input from the experimenter. On the other side, the current approach relies on common knowledge about the application and the separation of the composite behavior into tasks. Thus, the DP setup can be rather straightforwardly implemented since it does not assume sophisticated or difficult to obtain prior knowledge. For example, it has been very easy to employ DP for the coordination of two similar (Maniadakis et al., 2016b), or heterogeneous robots (Maniadakis et al., 2016a) in different application domains.

**Table 3:** Agents' time and error level for each action.

**Action**	**Action**	**Human**		**NAO**		**JACO**	
**Code**	**Name**	**Time**	**Efficacy**	**Time**	**Efficacy**	**Time**	**Efficacy**
A1	Message: “Clean the table”	[1,2]	9	[1,2]	9	[1,2]	9
A2	Wipe the table	[122,197]	9	NaN	1	NaN	1
A3	Message: “Thank you”	[1,2]	9	[1,2]	9	[1,2]	9
A4	-Rest	[1,2]	9	[1,2]	9	[2,3]	9
B1	Move to fruit shelf	[3,5]	9	[42,57]	7	NaN	1
B2	Wait bowl filling	[14,93]	9	[14,93]	7	[14,93]	9
B3	Move to the table	[3,6]	9	[33,48]	7	NaN	1
B4	Deliver breakfast	[1,3]	9	[13,25]	7	[11,15]	9
B5	-Rest	[1,2]	9	[1,2]	9	[2,3]	9
C1	Grasp fruit1	[1,3]	9	[38,56]	3	[11,15]	7
C2	Pick fruit1	[1,2]	9	[11,15]	5	[6,8]	9
C3	Place fruit1 in bowl	[2,4]	9	[42,73]	3	[12,19]	9
C4	-Rest	[1,2]	9	[1,2]	9	[2,3]	9
D1	Grasp fruit2	[1,3]	9	[38,56]	3	[11,15]	7
D2	Pick fruit2	[1,2]	9	[11,15]	5	[6,8]	9
D3	Place fruit2 in bowl	[2,4]	9	[42,73]	3	[12,19]	9
D4	-Rest	[1,2]	9	[1,2]	9	[2,3]	9
E1	Grasp fruit3	[1,3]	9	[38,56]	3	[11,15]	7
E2	Pick fruit3	[1,2]	9	[11,15]	5	[6,8]	9
E3	Place fruit3 in bowl	[2,4]	9	[42,73]	3	[12,19]	9
E4	-Rest	[1,2]	9	[1,2]	9	[2,3]	9

As discussed above, 20 different users have been involved in the real-world experimentation with the JACO and NAO robots, in order to assess the capacity of the composite time-informed system to support sHRI. For each user, DP is fed with the three breakfast items he will most likely be interested in at the given day, as they are predicted by the episodic memory inferencing. Accordingly, in the actual DP plan, fruit-1, fruit-2, and fruit-3 are substituted by the actual fruits to be served, i.e., kiwi, orange, banana, and so on. Moreover, the GTM-based estimate of human completion time and human efficiency on the table cleaning task is used as real-time input into the planner in order to actively adapt synchronization of the three agents.

**Figure 9:**
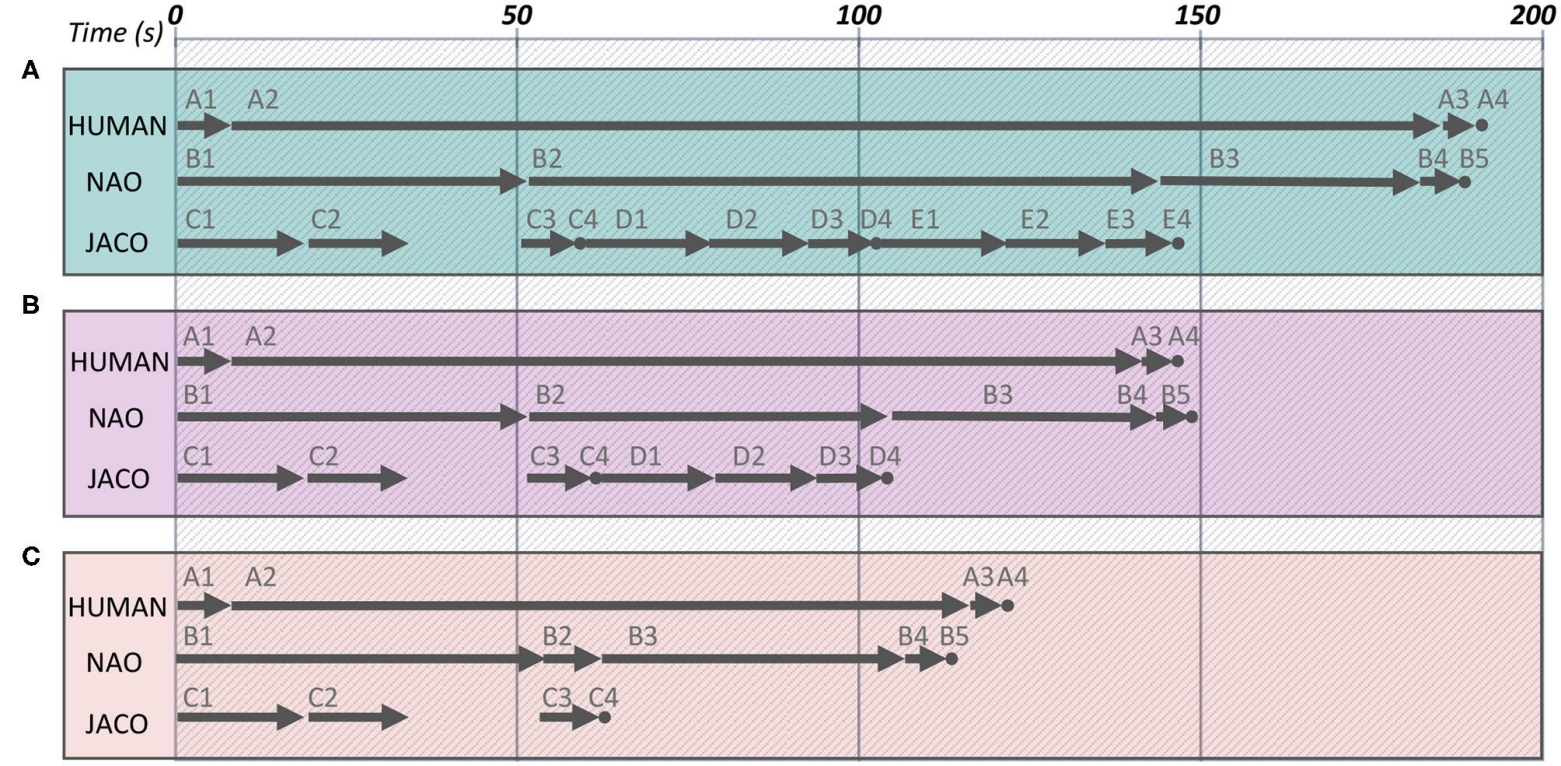
Indicative time distribution of individual tasks for breakfast serving. **(A)** Inattentive group, **(B)** Normal group, and **(C)** Dedicated group. Rest actions, of approximately 1 s, are marked with dots.

Graphical representations of task and action execution in different experimental sessions—one for each human profile—are depicted in Figure 9. In particular, Figure 9A summarizes interaction with user 4 of Table 1, who exhibits inattentive performance. As shown in the figure, the JACO robotic arm grasps and holds the first fruit (actions C1, C2), waiting the humanoid to arrive in a reachable area. As soon as the humanoid stops navigation (action B1), it waits for fruit filling, taking care of balancing issues (action B2). The robotic arm places the fruit it already holds in the bowl (action C3) and rests (action C4) waiting further instructions by the planner. The slow performance of the inattentive user provides enough time for JACO to add two more fruits in the humanoid's bowl (actions D1–D4 and E1–E4). As soon as all fruits are placed in the transfer bowl, NAO navigates toward the human (action B3), to deliver breakfast fruits (action B4) and rest (action B5). Almost the same time, the human completes cleaning (action A2), he gets fruits (action A3), and he is ready to enjoy breakfast (action A4).

Human-robot interaction in the case of user 11 who exhibits a normal performance profile is summarized in Figure 9B. The procedure followed is similar to the one summarized above, but now there is time for two fruits to be added in the bowl (actions C1–C4, and D1–D4). The humanoid robot delivers the fruits with a short delay of 2 s. The unfolding of multi-agent collaboration for the given user can be visualized in high resolution at https://youtu.be/1v4r0Xj8SF8.

Finally, Figure 9C summarizes the case of user 16 of Table 1, with an Dedicated performance profile. The high efficiency of this particular user is identified by the GTM, which informs accordingly the DP. The latter foresees that there is enough time for only one fruit to be added in the humanoid's bowl by the robotic arm (actions C1–C4). Immediately after that and in order to avoid delivery delay, the humanoid is instructed to navigate to human (action B3). The fruits are delivered on time and the user is ready to enjoy breakfast.

#### 5.3.4. User Satisfaction

To obtain insight on the users' view on the experiment we used a post-trial questionnaire consisting of five Likert statements assessed in the scale “strongly disagree,” “weakly disagree,” “neutral,” “weakly agree,” and “strongly agree.” More specifically, the following five Likert statements are examined to reveal user satisfaction:

**Q1—User satisfaction**: “The robots have delivered the right breakfast.”
**Q2—User satisfaction**: “The robots have delivered breakfast at the right time.”
**Q3—Human oriented performance**: “The implementation of robot tasks was adapted to my performance.”
**Q4—Performance expectancy**: “Robots performed better than expected.”
**Q5—Attitude toward using technology**: “Time informed interaction is crucial for domestic robot applications.”

Immediately after the experiment participants are informed that the current study is focused on time-aware multi-agent interaction. Then, they are asked to provide their individual opinions on the success of the system by filling out the aforementioned questionnaire. The aggregated results of participant responses on each question are summarized in Table 4. Clearly the participants expressed a positive opinion on the success of the experiment and the performance of the composite system.

**Table 4:** Overview of the responses provided by participants in Q1–Q5.

**Response**	**Q1**	**Q2**	**Q3**	**Q4**	**Q5**
Strongly disagree	0	0	0	0	0
Weakly disagree	0	0	2	0	0
Neutral	3	4	5	3	2
Weakly agree	9	6	9	11	4
Strongly agree	8	10	4	6	14

A one-way MANOVA revealed a significant multivariate main effect for the type of participants, Wilks' λ = 0.354, *F*_(4, 15)_ = 6.82, *p* < 0.002. This has been further confirmed by examining MANOVA nova separately for each participant type. In particular, statistically significant effects have been revealed for Inattentive Wilks'λ = 0.253, *F*_(4, 15)_ = 11.063, *p* < 0.0002, for Normal Wilks' λ = 0.233, *F*_(_4, 15) = 12.315, *p* < 0.0001 and for Dedicated Wilks' λ = 0.356, *F*_(4, 15)_ = 6.756, *p* < 0.002. The above indicate that the answers provided by participants to Q1–Q5 are affected by their own performance on the task.

The comparative study of the answers' means revealed significant statistical difference in the satisfaction of the Dedicated and Inattentive groups, while none of them was significantly different in comparison to the Normal group. In particular, the comparison showed that the system makes the Dedicated users more satisfied than the Inattentive ones, which is due to the current parameterization of the system targeting the minimization of robot delivery delay. The latter, i.e., the robots' task execution speed, is the factor which mostly affects the overall user experience, and is yet another strong indicator of the significance of “time” in sHRI sessions.

### 5.4. Quantitative Assessment

To proceed in the detailed quantitative assessment of the system, we have implemented a simulation environment which assumes simulated humans to interact with the two robots. We explore system performance in a broad range of situations and user profiles, by assuming three sets of 200 simulated users, each in accordance to the Inattentive (L), Normal (N), and Dedicated (A) profiles. In particular, to obtain GTM functionality within the simulated environment we use the recorded table cleaning data from real-users as they are classified in the three user profiles. To develop more “simulated” users, each data set is scaled by α%, with α randomly specified in the range [−10, 10]. Thus, for each simulation run, a real human data set is randomly selected from the corresponding group L, N, or A and scaled to represent the simulated human behavior.

In close association to the unfolding of the scenario in the real world, the experiment assumes the GTM to provide DP with estimates of the hypothetical human table-cleaning duration. Subsequently, the planner attributes tasks to the agents involved in the scenario. The agents implement their tasks as indicated by the user profile and the parameters of the given run. In order to satisfy temporal constraints, the generated plans are adapted online incorporating the latest GTM estimates of the human cleaning behavior. The scenario completes with the delivery of the breakfast menu to the simulated human.

**Figure 10:**
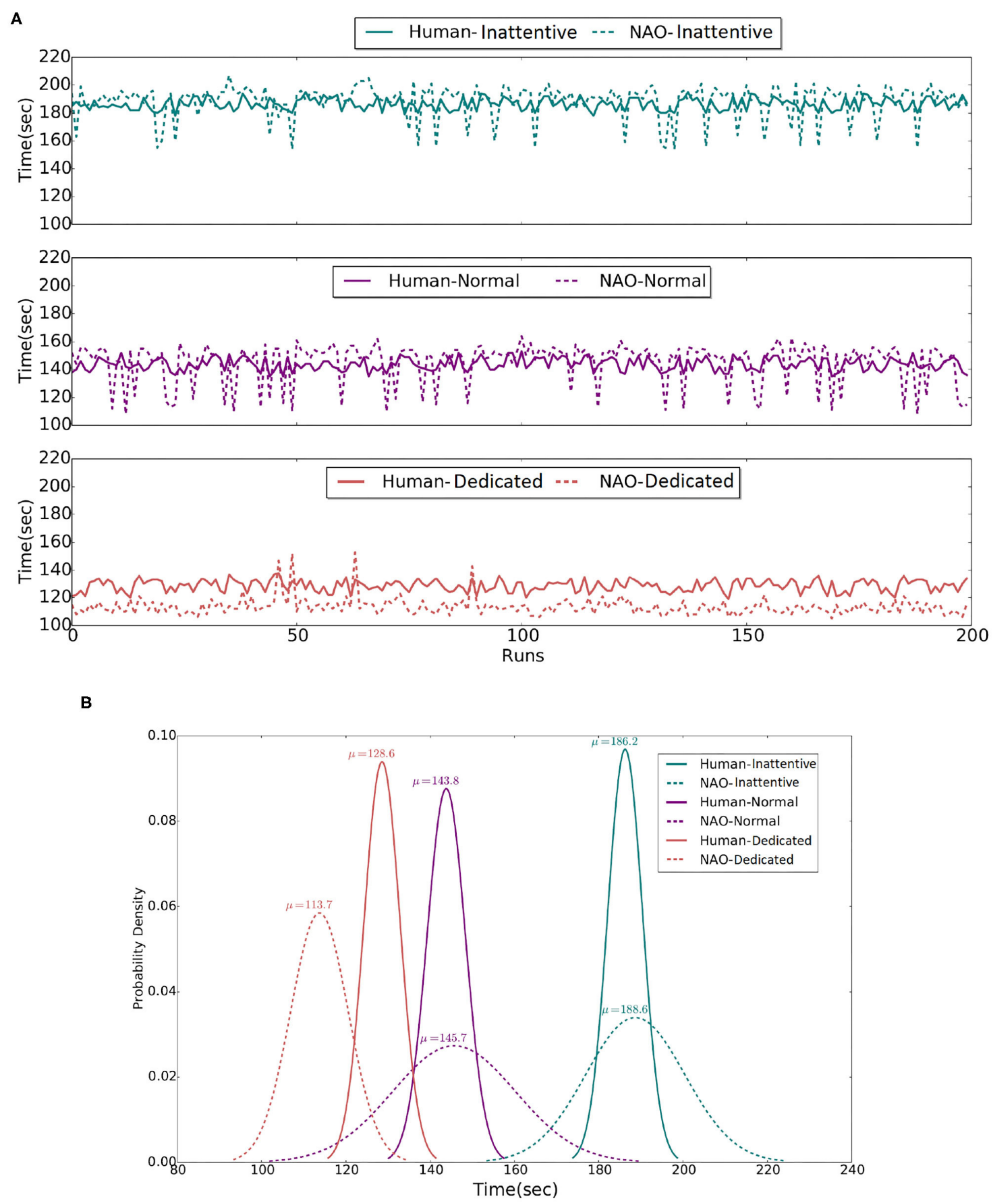
**(A)** Actual times for Human-agent (lines) and NAO robot (dotted-lines) over 200 runs for the three groups. **(B)** Gaussian distributions of actual times for Human-agent (lines) and NAO robot (dotted-lines) over 200 runs for the three groups. Green, purple, and red lines indicate the Inattentive, Normal, and Dedicated group, respectively.

The completion times for the table cleaning and breakfast delivery tasks (by the human and the NAO robot, respectively) for the 200 simulated runs are depicted in Figure 10A. The three plots correspond to the L, N, A user profiles assumed in the simulation runs. It is noted that NAO's duration is strongly biased by the number of fruit items to be served to the human. More specifically, each time JACO grasps a fruit to be added in NAO's bowl it needs approximately 35 s. This explains the quantization of NAO's completion time in approximately 188 s when delivering three fruits, 146 s when delivering two fruits, and 113 s when delivering only one fruit. According to the results, in the case of the inattentive participant three items are usually served (92% three items, 8% two items). In the case of the normal human behavior two fruits are typically delivered (83% two items, 17% one item). Finally, in the case of dedicated participants only one item is commonly served (98% one item, 2% two items). This is because of the strict time constraints and the need to complete fruit delivery prior to the completion of the table cleaning task by the human.

Overall, according to Figure 10A, in the case of normal and inattentive users, both humans and robots have similar completion times. In 65.0% of the runs in the case of inattentive user the robot is slightly delayed in comparison to the human. As later explained (see Figure 10B) the relevant delays are very short. The same is also true in 72.5% of the runs in the case of normal human behavior. This is because the robots give priority on fetching more fruits undertaking the risk of a very short delay in the delivery of the breakfast. Turning to the case of the dedicated human profile, the robots generally complete their task much earlier than the human, as assumed by the rather strict request to deliver breakfast prior to table cleaning. In only 4 out of the 200 runs the robot is delayed in comparison to the human. A close look on the unfolding of the 4 mentioned runs shows that delays are introduced due to the occasionally very fast navigation of NAO close to JACO which makes the planner believe there is enough time for placing two breakfast items in NAO's bowl, which actually proves that is not the case.

Figure 10 summarizes the behaviors described above. As indicated by the relevant Gaussian distributions the implement environment.

#### 5.4.1. Performance Metrics

To obtain insight on system's performance in association to the objective of fluent sHRI, the obtained results are quantitatively assessed using three metrics namely inter-module synchronicity, HRI synchronicity, and human idle time.

**A. Inter-module Synchronicity**. A key measure for assessing the effectiveness of the proposed framework regards its ability to exploit the time available for robot action, as it is predicted by the GTM. In the examined scenario this regards spending the predicted available time for fetching and transferring the maximum number of fruits to the human.

We introduce the inter-module synchronicity metric *e*_*im*−*sync*_ which aims to reveal the temporal coupling of system modules by contrasting the expected time of tasks implementation to the actual time spent. The metric is defined as follows:

(5) $$\begin{array}{rc} e_{im-sync}^{x} & =1-𝔼\left\{\frac{\left|t_{H,p}^{x}-\max\left(t_{H}^{x},t_{N}^{x}\right)\right|}{\max\left(t_{H}^{x},t_{N}^{x}\right)}\right\} \end{array}$$

where *x* ∈ {*L, N, A*}, $t_{H,p}^{x}$ is the early prediction of human completion time and $t_{N}^{x}$, $t_{H}^{x}\in {\mathbb{R}}^{200}$ are the actual completion times of NAO robot and human agent, respectively, for the total of 200 simulation runs.

The obtained results are summarized in Table 5 (Cols. 2-3). The high accuracy and low uncertainty values observed for all three human profiles are explained by the largely accurate predictions by the GTM module and the inherent flexibility of the collaboration plans developed by DP, which are sufficiently adapted to the actual implementation and the temporal characteristics of multi-agent synergistic performance.

**Table 5:** Performance metrics evaluation.

	**Inter-module synchronicity**		**HRI synchronicity**		**Human idle time**	
**User profile**	**μ(*e*_*im-sync*_)(%)**	**σ^2^(*e*_*im-sync*_)(%)**	**μ(*e*_*hri-sync*_)**	**σ^2^(*e*_*im-sync*_)**	**μ(e_*h-idle*_)**	**σ(e_*h-idle*_)**
Inattentive	98.3	0.07	2.4	126.34	5.7	31.7711
Normal	96.8	0.12	−1.9	174.72	6.3	32.1652
Dedicated	94.1	0.04	−14.8	52.38	0.3	5.5752

**B. HRI Synchronicity**. The current metric focuses on the synchronicity of the composite artificial system with the real world. In particular, the metric describes how well human and robotic activities are synchronized, that is what is the average time that one side has to wait for the other. Synchronicity, ***e***_*hri*−*sync*_, is defined as follows:

(6) $$\begin{array}{r} e_{\text{hri-sync}}^{x}=t_{N}^{x}-t_{H}^{x} \end{array}$$

where symbols are as above. As witnessed by the HRI synchronicity values shown in Table 5 (Cols.4-5), the robotic agents are effectively synchronized with the ongoing procedures of the external environment. According to the same table, the less accurate synchronization is observed in the case of the dedicated user profile, which clearly indicates that, for the given profile, the system prefers to complete earlier the robotic task in order to minimize the risk of a possible human waiting. For the other two profiles, the robotic agents are quite accurately synchronized to humans.

The relevant observations are also reflected to the particularly low idle time of dedicated humans, which increases in the case of the Normal and Inattentive profiles (second plot of the figure).

**C. Human Idle Time**. To enhance human experience in sHRI sessions, the implemented system should ideally minimize the human waiting time and thus improve the responsiveness of the composite system to human requests. Similar to Hoffman (2013), we use the *Idle Time* metric to assess system performance. In particular, the human idle time $e_{\text{h}-\text{idle}}^{x}$ is defined as follows:

(7) $$\begin{array}{r} e_{\text{h}-\text{idle}}^{x}=\max\left(t_{H}^{x},t_{N}^{x}\right)-t_{H}^{x} \end{array}$$

where symbols are as above. As shown in Table 5 (Cols.6-7) in the case of inattentive and normal user profiles, there is a short human waiting which averages to 5.7 s in the former case and 6.3 s in the latter. However, in the context of human daily activities, durations in the range of few second are typically considered very small and do not annoy humans. In the case of the dedicated user profile, the robotic tasks typically complete earlier than the human. The only 4 out of 200 cases that robots get delayed result into the average of 0.3 s human idle time, which is particularly low and satisfactory for humans.

## 6. Conclusions

The long-term symbiotic interaction between humans and robots has tremendous potential as the robots bond with people, and can significantly affect humans' daily activities. Despite the increasing research endeavors devoted to human-robot synergies, we still know little about building systems that function smoothly and effectively within the context of prolonged companionships.

The integration of “sense of time” into a robotic system is at the core of a fluent sHRI, since it traverses almost every aspect of the relevant interactions. In this work, we examined the role of time focusing on three major disciplines of human-robot interaction: (i) past episode storage and experience-based inferring, (ii) activity duration prediction and human efficiency estimation, (iii) multi-agent coordination for synergetic action planning. The integrated performance of the relevant modules (i) implements time-inclusive criteria to match (assign) tasks to agents, coordinating heterogeneous agents to perform as an effective team, (ii) combines user information referring to different temporal granularities by blending the long-term, memory-inferred user preferences with the short-term, real-time predicted user expectations, (iii) monitors the dynamic scenario progress in real time to support the situated adaptation of multi-agent interaction, thus being particularly useful in sHRI scenarios.

Each module (past/episodic memory, present/Generative Time Models, future/daisy planner) benefits from the use of time-informed symbolic representation of past sHRI episodes facilitates highly accurate predictions about future scenario unfoldings. Our ongoing and future work focuses on the advancement of the individual modules in the directions shortly summarized below:

- Episodic Memory. The analysis of past data may provide significant insight into the needs and preferences of the individual users. In this direction we are currently considering mechanisms to exploit real-time robot experiences that are stored in memory, through off-line (e.g., night-time) training and dynamic querying mechanisms that recall or infer information about the users to provide accurate and personalized predictions. At the same time, research endeavors aim to improve the efficiency of the episodic memory module itself, either by fully automating the lifecycle—i.e., update, merge or forget with respect to the importance factor—of each stored memory, or by integrating the capacity to represent and exploit higher level concepts, such as the knowledge or behavioral expertise of different users.
- Generative Time Model. Given that accurate estimates of the duration of human actions greatly facilitates fluency in HRI, the current work puts forward the association of the temporal primitives of actions with the task progress. A GTM is employed to analyze human performance and provide robust estimates about the temporal aspects of the observed activity. Future work will extend the framework to discrete primitives, in order to provide a holistic methodology on temporal predictions and will consider comparative productivity measurements with emphasis on fatigue detection.
- Daisy Planner. The orchestration of team members considering their individual skills and limitations has been beneficial for sHRI applications. Our future work focuses on the temporal interruption of task implementation to enable the adaption of robot behavior to urgent un-predicted circumstances. Early work in this direction has showed that to sufficiently address this issue, the DP must distinguish between tasks (petals) which, when interrupted, can be resumed from where they left off, and tasks which, when interrupted, must be carried out completely from the beginning. Another promising direction regards the implementation of a hierarchical daisy representation of tasks, thus extending the span of human-robot interaction from the range of minutes to the range of hours, including also the ability to merge tasks similar to Stock et al. (2015).

Beyond improvements on the three core modules, future work regards the exploitation of existing computational models of human time perception (Maniadakis and Trahanias, 2012, 2016b), the time-informed kinesthetic teaching of robots (Koskinopoulou et al., 2018, 2019), and how sense of time interacts with emotions, a rather unexplored direction that has the potential to significantly improve sHRI (Maniadakis et al., 2017).

Moreover, it is of particular importance to explore the usability of the system in HRI setups that involve more agents (both robots and humans). Along this line, planned experiments will mainly focus on teams with dynamic synthesis (i.e., humans may freely enter or leave the team) and more complex, natural interaction setups with non-fully scripted scenario evolution. Overall, we envision robotic systems that greatly capitalize on temporal cognition to seamlessly integrate into the heavily time-structured human societies.

## Data Availability Statement

The datasets generated for this study are available on request to the corresponding author.

## Ethics Statement

The studies involving human participants were reviewed and approved by FORTH Ethics Committee (FEC). The patients/participants provided their written informed consent to participate in this study.

## Author Contributions

All authors have equally contributed to the submitted work.

## Conflict of Interest

The authors declare that the research was conducted in the absence of any commercial or financial relationships that could be construed as a potential conflict of interest.

## Footnotes

^1^The subtraction of fuzzy numbers is as follows: (*a*_1_, *a*_2_, *a*_3_, *a*_4_) − (*b*_1_, *b*_2_, *b*_3_, *b*_4_) = (*a*_1_ − *b*_4_, *a*_2_ − *b*_3_, *a*_3_ − *b*_2_, *a*_4_ − *b*_1_).

^2^ROS OpenSlam Gmapping http://wiki.ros.org/gmapping

^3^ROS Adaptive Monte Carlo localization, https://github.com/ros-planning/navigation

^4^ROS move_base http://wiki.ros.org/move_base

^5^ROS cmvision_3d https://github.com/OSUrobotics/cmvision_3d
